# Effect of Processing Conditions of Enzymatic, Extrusion, and Hybrid Treatment Methods on Composition and Selected Technofunctional Properties of Developed Wheat Flour

**DOI:** 10.1155/ijfo/3317924

**Published:** 2025-05-27

**Authors:** Piotr Lewko, Agnieszka Wójtowicz, Michał Rudaś

**Affiliations:** ^1^Department of Food Process Engineering, University of Life Sciences in Lublin, Lublin, Poland; ^2^PZZ Lubella GMW Sp. z o.o., Lublin, Poland; ^3^The Central Laboratory of Research, University of Life Sciences in Lublin, Lublin, Poland

## Abstract

In this study, wheat flour characterized by a high content of nonstarch polysaccharides was fortified with enzymes and then subjected to low temperature (up to 85°C) extrusion cooking treatment. Conventional enzymatic hydrolysis with cellulase and cellulase–xylanase blend, as well as extrusion and hybrid enzymatic–extrusion treatments, was tested under variable conditions. Extrusion of wheat flour was applied at 23%–27% initial moisture at the temperature range of 40°C–80°C. Proximate composition, polysaccharide content, and its fractions, as well as rheological and technofunctional properties, were tested. Extruded and hybrid-modified wheat flour showed a significant decrease in fat, ash, insoluble fiber content, gelatinization beginning temperature, dough stability, starch gelatinization, amylase activity, starch retrogradation, and gluten performance index, whereas increased hydration capacity, max viscosity, setback, protein weakening, and solvent retention capacity were evidenced in the presence of all tested solvents. Soluble and insoluble fractions of nonstarch polysaccharides were, however, significantly different, especially if the hybrid cellulase–xylanase–extrusion method was applied to wheat flour. Moreover, the crystalline structure of wheat flour changed significantly after extrusion and hybrid treatments. In addition, the microstructure showed a significant agglomeration of the extruded flours due to starch gelatinization and formation of melted phase in all extruded and hybrid-treated flours, with visible fibrous particles coming from the outer layers of wheat grains as polysaccharide fractions. Extruded wheat flour, characterized by increased viscosity, hydration, and solvent retention ability, can be used as a “clean label” improver in mixtures for various bakery products, especially bread.

## 1. Introduction

The extrusion cooking process is increasingly used in many fields of food, feed, or biopolymer processing [1, 2]. Controlling the effect of the extrusion cooking conditions on the changes in basic and functional components, as well as physical properties and texture, is still a big challenge [3, 4]. The inclusion of health-promoting and functional components and valuable byproducts may easily improve the nutritional potential of the extruded products [5, 6]. The extrusion process has been studied for decades for its potential in the processing various cereals or plant-based products. Extrusion processing brings about protein and starch structure changes and gelatinization of starch granules [7, 8] and is widely used to develop infant foods, snack foods, ready-to-eat breakfast cereals, modified starches, meat analogues, pet foods, among other products [9–15]. During extrusion, thermomechanical action can cause denaturation of proteins and change their structure and solubility due to the combined action of shear forces, heat, pressure, and oxygen [16, 17]. Extrusion of flours produces gelatinized, melted, or fragmented starch with increased damaged starch content, together with a reduction in lipid oxidation due to inactivation of enzymes, an increase in soluble fiber, protein fragmentation, and a possible reduction in thermolabile vitamins, antinutritional factors, and microbial load [1]. The disruption of the starch granules by gelatinization occurring during extrusion also makes starch more accessible and susceptible towards enzymatic hydrolysis afterwards—leading to a more intensive solubility [18]. These changes allow for the adjustment of its rheological and hydration properties in response to arising needs demanded by new food trends. Extruded wheat flours may therefore be an interesting alternative to chemically pregelatinized (hydroxypropylated or crosslinked) starches or hydrocolloids in the bakery industry. Extrusion cooking has been widely reported in the processing of bran to change the rheological and chemical characteristics of fibrous fractions, for example, in wheat bran [19, 20], corn bran [21], and rice bran [2, 22]. Blasting extrusion processing (BEP) has also been developed so as to modify wheat bran by enabling partial disruption of its cellulose and hemicellulose, as well as to bring about the release of its soluble saccharides from its original continuous fiber matrix [23].

Some researchers have attempted to apply combined or hybrid methods together with extrusion cooking to modify selected components by the addition, among others, of specific chemicals [18, 21, 24, 25], microwaving [26], ultrasound treatment [27], or enzymatic digestion [22]. Zhou et al. [18] investigated ethanolic-assisted extrusion of cornstarch and reported the destruction and reorganization of the crystalline structure during extrusion, with a significant positive effect of ethanolic extrusion to obtain cold water-swelling starch. Enzymatic extrusion is a quite new method, in which the extruder is used as a continuous bioreactor or enzyme reactor to accelerate the enzymatic reaction [28]. Enzymatic-supported extrusion processing can effectively treat complex biopolymers with a high degree of polymerization, crystallinity, and structural strength and can also impart a porous microstructure and expose the enzyme reactive site [2]. Some researchers have employed enzymatic–extrusion to modify cereal bran or fiber-rich fractions [29]. Dang and Vasanthan [22], for example, found that the application of both enzyme treatment and extrusion improved the solubility of rice bran dietary fiber and other soluble components, especially as the sequential extrusion–enzyme treatment significantly increased the total soluble pentosan content, compared to individual or simultaneous treatments. Kong et al. [30], in turn, tested the effect of comodification by extrusion and enzymatic hydrolysis (with cellulase, xylanase, high-temperature *α*-amylase, and acid protease) on water-extractable arabinoxylan (AX) and physicochemical properties of black wheat bran. Depending on the type of enzymes, its level and activity or additional processing, and its treatment order, with regard to wheat flour, its functional polysaccharides may be diversely modified.

The use of various enzymes in baking is widespread in industrial solutions. They are responsible for improving the machinability of the dough and the quality of the bakery product. Significant improvements can be obtained by adding enzymes to flour due to slowing down of the crumb firming. Among others, these include increase in bread volume, improvement in crispness and color of the crumb crust, better sensory characteristics, and increased shelf-life of products [31]. Different types of enzymes can be applied to change the selected fractions of cereals and plants, that is, as proteases to hydrolyze rice proteins [32], amylases to enzymatic saccharification of various starch origins [33], or cellulase and hemicellulases to fibrous fraction modification [34]. At present, amylase, cellulase, and protease are used in enzymatic–extrusion playing a role in liquefying starch, producing bioethanol, and improving dough quality [2]. Cellulases are extremely important enzymes both industrially and in the natural world, because they play a major role in the global carbon cycle by degrading insoluble cellulose to soluble sugars. Xylanase, for example, are cellulase enzymes used in the food industry in the bakery sector to enhance the stability of dough, to generate a softer and uniform crumb structure, and to increase the specific volume of breads. Through the action of xylanase, the contained water becomes distributed from the pentosan phase to the gluten phase, thus amplifying volume via increased extensibility of the gluten due to the rise in gluten volume fraction. Additionally, xylanases have been discovered to delay staling, improve the texture of high fiber bread, and balance the variable quality of flour used to bake wheat bread. Xylanases are required to act collectively to hydrolyze xylans (e.g., AXs and glucuronoxylans) due to the structural heterogeneity and complexity of xylans and the specificities and modes of catalytic action of xylanases [35, 36].

Nonstarch polysaccharides (NSPs) and AXs are interesting cereal components. These are cereal fiber compounds found predominantly in the cell walls of the endosperm and aleurone layer in cereal grains. Among the cereal grains (and grinding processes), AX content differs significantly at 7.6%–12.1% in rye, 4.8%–7.6% in wheat, 4.4%–8.1% in barley, and 1.7%–2.7% in oat [37]. AXs are an important dietary fiber source due to their contained functionalities and health benefits, including cholesterol lowering activity, fecal bulking effect, prebiotic effects, high antioxidant activity, vitamins, minerals, and the presence of other bioactive components [36, 38, 39]. Kaur et al. [40] reported two main types of AXs: water extractable arabinoxylans (WE-AXs) (one-third) and water unextractable arabinoxylans (WU-AXs) (two-thirds). These are characterized by different compositions, as well as action to water and functionality, for example, WE-AXs are semiflexible and highly soluble in water due to high glucose and arabinose substitution; they stabilize gas pores, slow down bread staling, and improve dough quality by increasing water absorption (Hyd) and bread volume, but WU-AXs are more rigid and insoluble due to the presence of cellulose and hemicellulose fractions [27]. The AXs are the major polymers present in the cell wall of wheat grain and exhibit different physicochemical properties (hydration properties, water solubility, and viscosity). Moreover, these properties or its structure may change through technological treatments.

The objective of our study was to evaluate the effects of enzyme addition, low-temperature extrusion cooking, and hybrid enzymatic–extrusion treatment on particular properties of developed wheat flour consisting of selected breaking, milling, reducing, and sifting passages. Treatments were selected as individual or hybrid, with various types and levels of enzymes (cellulase and xylanase) under variable extrusion conditions. Nontreated native flour (F), enzyme-fortified flour (FC and FCX), and extruded samples treated without (EF) or with the presence of enzymes (EFC and EFCX) were tested for proximate chemical composition, NSP and AX contents and composition, pasting properties, and rheological and technofunctional characteristics, as well solvent retention capacity (SRC) and gluten performance. Structural changes were observed via X-ray diffraction (XRD) analysis and on the basis of microscopic pictures taken via scanning electron microscopy.

## 2. Materials and Methods

### 2.1. Raw Materials and Enzymes

New flour blend (F) from common wheat of the Laudis variety was employed for the tests. This consisted of blends subject to selected breaking, milling, reducing, and sifting passages, composed in accordance with our previous research [41] as a raw material suitable for the production of wheat bread, characterized by an average gluten content of 31%, ash 0.72%, descent time 340 s, and average Hyd 60%. Commercially available baking enzymes were used to fortify the flour (the amount of enzyme being determined based on preliminary tests and suggestions of the enzyme manufacturer). These included: Bakezyme WholeGain—cellulase from *Trichoderma reesei* (DSM Food Specialities B.V., Delft, The Netherlands) with declared enzyme activity 1475 EGU/g, and VERON 292—xylanase from *Aspergillus niger* (AB Enzymes GmbH, Darmstadt, Germany) with declared enzyme activity min 1701 XylH/g. Two combinations of enzymatic modification were investigated: the Bakezyme WholeGain cellulase enzyme was used in an amount of 120 ppm (samples marked C), and the mixture of Bakezyme WholeGain cellulase and VERON 292 xylanase were employed in amounts of 60 ppm and 50 ppm, respectively (samples marked CX). Wheat flour was fortified with these enzymes by gentle mixing of flour with dry enzymes in a powdered form and left for 0.5 h in a room temperature to initiate the enzyme activity. The fortified flours were then tested (FC and FCX) or subjected to extrusion processing under variable conditions. The experimental design with selected variables and coded samples is presented in Table 1.

### 2.2. Extrusion Process

The treatments of flour without (EF) or with different enzyme combinations (EFC and EFCX) were carried out by extrusion cooking processing using a corotating twin screw extruder Evolum25 (Clextral, Firminy, France) equipped with double screws 25 mm of diameter each, configuration of *L*/*D* = 24 and forming die with a single open 3 mm in diameter. Twin screw configuration consisted of 32 modules on one grooved shaft, with the total length of 24 D, with 3.75D supply modules, 12.5D transport modules, 1.5D mixing elements, 2D mixing with groove elements, 4D compression modules, and 0.25D interface ring, set in a certain order. Feeding rate 10 kg/h was kept constant by a Brabender volumetric gravity feeder. Screw speed was set at 400 rpm throughout the experiments. The level of feed moisture was set to 23%, 25%, and 27% as calculated based on the initial moisture of flour tested according to the AACC 44-15.02 method [42], while water in proper amounts (1.2, 1.5, and 1.8 L/h, respectively) was pumped directly to the second zone of the extruder barrel by a connected volumetric water pump. During the extrusion process, the temperatures in individual barrel sections, the product temperature, and the working pressure inside the barrel were monitored. The extruder utilizes electrical resistance heaters to create six heating/cooling zones with electrical resistance heaters monitored by thermocouple sensors. Processing temperature was set as follows: 40°C/50°C/60°C/65°C/70°C/80°C, forming die temperature set at 85°C, product temperature measured 82°C–95°C (increasing with lower feed moisture), and pressure on the die measured 80–102 bar (decreasing with higher feed moisture). Extrudates were cut with a two-blade knife working with speed of 300 rpm. Samples were collected when the extruder was working stably. The collected samples obtained after extrusion were dried in a laboratory shelf dryer at 40°C to reach the moisture content below 12% to ensure safe storage. Extrudates were ground to the particle size below 500 *μ*m in a laboratory grinder (TestChem, Radlin, Poland) and stored closed for further analyses. The effects of treatment conditions on selected characteristics were tested on dry flour, diluted flour suspension, or on the dough matrix to compare the physiochemical characteristics of modified flours.

### 2.3. Proximate Chemical Composition

Proximate chemical characteristics were determined according to international standards: 44-15.02 AACC method for moisture, 46–10 AACC method for protein (Nx6.25), 30–10 AACC method for fat, and 08–01 AACC method for ash contents [42]. The 991.43 AOAC enzymatic–gravimetric method [43] was used to appoint the content of total dietary fiber (TDF) as well as soluble dietary fiber (SDF) and insoluble dietary fiber (IDF) fractions.

### 2.4. NSP and AX Profiles

The NSP content was assessed by gas chromatography as claimed by Englyst and Cummings [44]. The total NSP (T-NSP) content is the amount of sugars: arabinose, xylose, mannose, galactose, and glucose according to 32-25 AACC standard procedure and 994.13 AOAC [42, 43, 45]. Performed analysis allowed to disengage soluble nonstarch polysaccharide (S-NSP) and insoluble nonstarch polysaccharide (I-NSP) fractions of NSPs and to indicate the qualitative and quantitative polysaccharide composition of both fractions. The total content of arabinoxylans (T-AX) as well as insoluble arabinoxylan (I-AX) and soluble arabinoxylan (S-AX) fractions were calculated based on the content of each fraction. Soluble and insoluble monosaccharide fractions were additionally detected in each fraction after acid hydrolysis. Then, the conversion of received hydrolysates into volatile alditol acetates was done. Some reagents were added to 1 mL of tested sample: 2 drops of 2-octanol, 0.26–0.28 mL of 12 M ammonia solution, and 0.1 mL of sodium borohydride solution in ammonia (100 mg BH_4_ in 1 mL of 3 M NH_4_OH). Incubation at 40°C for 40 min was followed by adding 0.1 mL of glacial acetic acid to the hydrolysate, mixing, adding 0.2 mL of 1-methylimidazole, and 2 mL of acetic anhydride to 0.2 mL of the collected sample. After 30 min of cooling, 4 mL of distilled water and 1.15 mL of dichloromethane were added to the prepared solution followed by 1-min shaking. The aqueous and organic phases were separated. Organic phase was fed to the autosampler of gas chromatograph (Autosystem XL, Perkin Elmer, Shelton, CT, United States), equipped with an Rtx-225 capillary quartz column (0.53 mm × 30 m), a flame ionization detector (FID), and a split injector. The temperature program of chromatograph column was as follows: 1 min in initial temperature at 185°C, increase to 215°C with the speed of 5°C/min, and 10 min in isotherm at 215°C. Analyses were performed at following operating settings: 275°C of injector and detector temperature and 2 mL/min flow and helium as carrier gas [46].

### 2.5. Rheological Properties of Dough

Rheological properties of dough prepared from the tested native and modified flours were measured using a Chopin Mixolab (Chopin Technologies, Villeneuve-la-Garenne, France). The Chopin+ flour protocol was applied with the following settings: 75 g of dough weight, 30°C of hydration water temperature, 80 rpm of mixing speed, and 45 min of total analysis time. In the first test phase, flour and water were added to obtain a dough consistency of maximum 1.10 Nm (± 0.05). A standard Mixolab protocol was applied at the following settings: 30°C for 8 min, heating with the speed of 4°C/min for 15 min, 7 min holding at 90°C, 10 min cooling to 50°C with the speed of 4°C/min, and 5 min holding at 50°C. Hyd, protein weakening (C2), starch gelatinization (C3), amylase activity (C4), and starch retrogradation (C5) were evaluated [47].

### 2.6. Pasting Properties

Pasting properties were measured by employing a Brabender Viscograph-E (Brabender GmbH & Co. KG, Duisburg, Germany) working at 700 cmg torque and 75 rpm according to the ICC 169 procedure [48]. Solution of 450 mL distilled water and 80 g flour (adjusted to 14% moisture content) was poured to the Viscograph-E canister plugged into the heating chamber with attached spindles. During the test, the slurry was heated from 30°C to 93°C with the speed of 1.5°C/min, 15 min held at 93°C, cooled down to 50°C with the speed of 3°C/min, and 15 min held at 55°C. Viscosity in Brabender units (BUs) was evaluated based on the resistance to stirring. The beginning and end of gelatinization temperature (°C), maximum viscosity (BU), trough viscosity (BU), final viscosity (BU), breakdown (max viscosity minus trough viscosity, BU), and setback (final viscosity minus trough viscosity, BU) were determined using the Viscograph-E software.

### 2.7. Hydration Properties

SRC was evaluated according to the AACC 56-11 approved method [42, 49]. SRC is the mass of solvent absorb and continue to hold by the swollen flour precipitate after centrifugation and is expressed as % of the original flour mass (adjusted to 14% moisture). The solvents used were as followa: Wa (deionized water), Su (50 wt% solution of sucrose in water), La (5 wt% solution of lactic acid in water), and Sc (5 wt% solution of sodium carbonate in water). Then, 5 g of flour sample was transferred to a 50-mL centrifuge tube and mixed with 25 g of solvent. Next, the sample remained to solvate for 20 min agitated periodically (every 5 min for 5 s). Then, the samples in tubes were placed in an Eppendorf 5702 centrifuge (Eppendorf AG, Hamburg, Germany) and centrifugated for 15 min at 2500 rpm (646 g force). Resultant supernatant was separated, and the tubes were left for 10 min. The sample was then weighed, and the following hydration properties were calculated: WaSRC (water retention capacity), SuSRC (sucrose solvent retention capacity), LaSRC (lactic acid solvent retention capacity), and ScSRC (sodium carbonate solvent retention capacity) [50]. Additionally, gluten performance index (GPI) was determined based on Rani et al. [49] according to the ICC method by dividing the obtained value of LaSRC by the sum of SuSRC and ScSRC values.

### 2.8. Structure Analysis With X-Ray Diffractometer

Powdered samples were conditioned by 3 days in a testing room at controlled temperature (19°C) and at a constant relative humidity (28%) and placed in an autosampler. The XRD method was applied utilizing a high-resolution Empyrean powder diffractometer (PANalytical, Almelo, The Netherlands) with Cu K*α*1 radiation (*λ* = 1.54178 Å). Samples were measured over a range from 5° to 70° at diffraction angle geometry *θ*–2*θ*, with a counting time of 400 s per data point and a step size of 0.01° [51]. Obtained results were baselined and differences in the structure were analyzed at specific peak angles. Crystallinity was determined using WAXFIT software [52]. All data were normalized, and the background was approximated by hyperbolic function. Data for F were fitted to a Gauss–Cauchy model containing 15 functions to describe the crystalline phase and 2 functions to describe the amorphous phase. Extruded flours' data were fitted to a Gauss–Cauchy model containing 13 functions to describe the crystalline phase and two functions to describe the amorphous phase. The degree of crystallinity (%) was calculated via WAXFIT software after fitting models and correcting backgrounds as the ratio of the area under the crystalline phase curves to the sum of the areas of the crystalline and amorphous phases [53, 54].

### 2.9. Microstructure Characteristic by SEM

The pictures of microstructure of native and modified flours were taken using a Vega Tescan LMU scanning electron microscope (Tescan, Czech Republic). Flour particles were attached to aluminum stands with silver double-sided adhesive tape and coated with gold spraying using the Emitech K550X Sputter Coater (Emitech, United Kingdom). The accelerating voltage of the SEM was 20 keV, with absorption currency of 9 pA. SME pictures were taken under high vacuum (7.5 × 10^−2^ Pa) with magnifications ×600 and ×2000 [9].

### 2.10. Statistical Analysis

All analyses were performed in triplicate, and results are expressed as average values ± standard deviation. One-way analysis of variance (ANOVA) followed by the Tukey's post hoc test to compare means was applied to analyze the obtained results at the 0.05 significance level with a Statistica 13.3 software (StatSoft, Inc., Tulsa, OK, United States). Results were combined into homogenous groups at significance level 0.05 indicated with similar letters in tables and figures. The correlation matrix with Pearson's correlation was assessed between all the tested properties, and significant coefficients were found using Statistica 13.3 software (StatSoft, Inc., Tulsa, United States) within the 95% confidence interval.

## 3. Results and Discussion

### 3.1. Proximate Composition

Proximate chemical composition of untreated, extruded, enzyme-fortified, and hybrid-treated wheat flours is presented in Table 2. Results are presented at moisture content varied from 13.76% to 13.90% in F and from 10.21% to 10.54% in extruded flours without significant differences between treatment conditions. The protein content of native wheat flour F was at 14.65%, and similar results were noted if fortification with enzymes was applied (14.52%–14.64%). Extrusion and hybrid enzymatic–extrusion treatments with combined CX blend increased or remain unchanged the protein content in the modified flours (except of EFC25 sample) as compared to native wheat flour F. As opposed to our results, Zhou et al. [36] found lowering the content of protein (9.85%) in xylanase-extracted destarched wheat bran and with alkali treatment (4.10%) against of 18.4% in native bran. Moreno-Rivas et al. [55] also found that xylanase addition had a reduction effect on the proteins solubility in extruded nixtamalized corn flour, probably by the interaction with AXs.'

We noted that fat content decreased significantly after extrusion treatment of flour, both without or with enzymes added. This is due to formation of lipid–starch complexes under extrusion conditions—as reported by many authors [1, 4, 12, 24]. Ash content in the tested samples do not differ significantly if flour was fortified with both enzymes (FC and FCX) and was lower than in samples after extrusion. Moreover, cellulase addition significantly lowered TDF (6.14%) in the untreated flour (FC), especially the SDF fraction (2.33%) as compared with F (6.80% and 2.86%, respectively). Extrusion without enzymes at variable feed moisture lowered TDF content (5.30%–5.53%), in both IDF (3.06%–3.79%) and SDF (1.83%–2.42%) fractions. When the hybrid enzymatic–extrusion method was applied, the fiber content in treated flour varied depending on processing conditions. Cellulase addition and the highest moisture (27%) increased the TDF content in the treated flour (7.12%), both soluble (2.93%) and insoluble (4.18%) fractions. The opposite effect was noted if enzymes blend CX was added to flour and then subjected to extrusion—here, the lowest feed moisture (23%) in sample EFCX23 significantly increased the formation of fiber (7.74%), especially the SDF fraction (4.12%) as compared to sample F. These high TDF values, especially the SDF fraction, are the result of the most effective combinations of processing conditions (water content level and enzymes used). Moreover, these samples were characterized by the presence of the highest content of NSPs, especially S-NSP and T-AX, as a result of the integrated enzymatic–extrusion treatment in EFC27 and EFCX23 samples (as presented in Table 3). The most effective way to increase the amount of soluble fractions in modified wheat flour was to use a combination of an enzyme complex and a low moisture extrusion processing (EFCX). The application of combined cellulase–endoxylanase complex is more efficient for extracting water-unextractable AXs than xylanase alone [56]. Under the hybrid treatment, the bonds of insoluble polysaccharides, as cellulose and hemicellulose, and the continuous fiber matrix were partially broken and released soluble saccharides after extrusion due to the combined effects of intensive shearing and enzyme action [20]. The lowest TDF was noted in EF samples extruded without enzyme addition without a strong effect of feed moisture. In almost all samples, content of IDF was lower after modification than in F; this may suggest that SDF is more sensitive to enzymatic or extrusion treatment [30]. The conversion of IDF to SDF might be the effect of high shear and high temperature during extrusion treatment which possibly breakdown the larger molecules, leading to a decrease in TDF and IDF contents, but at the same time an increase in solubility of fibers [19]. Additionally, enzymes, like cellulase or xylanase, can modify SDF content and enzyme-assisted extrusion was found as an effective treatment to increase oligosaccharide soluble fractions, for example, in rice bran [2]. Ye et al. [57] tested various methods of wheat bran treatment and they reported fermentation and extrusion effects on increasing the soluble dietary fiber content in processed bran. Accordingly, extrusion treatment may increase fiber solubility as we noted that the water-binding capacities of extruded brans were lower than that of nonextruded wheat bran [58]. The results presented by Aktas-Akyildiz et al. [19] confirmed increased SDF content in bran samples subjected to enzymatic hydrolysis. Extrusion treatment conditions, especially the screw speed as the most effective parameter, showed that extrusion can disrupt the wheat bran microstructure and thus increase the soluble fiber content. Similar observations in improvement of dietary fiber solubility during extrusion of wheat bran were noted by other authors [58–60]. In our study, not bran, but developed wheat flour [41] was used in the experiment, so the results may be different due to the higher presence of starch and proteins in the flour. As stated by Alam et al. [1], these are the components responsible for the formation of complexes and starch gelatinization under extrusion, even if low-temperature processing is applied.

### 3.2. Insoluble and Soluble Polysaccharide Characteristics

The content and fractions of polysaccharides are very interesting with regard to their nutritional and technological roles. Cereal bran is rich in NSPs, mainly hemicellulose, including AX and *β*-glucan, as well as cellulose and lignin [36, 40]. Among these NSPs, AXs are important dietary fiber components. Wheat AXs are present in endosperm (3%–5% of total endosperm), aleurone, and bran cell walls (approximately 60%–70% of the entire cell wall) [39]. If flour is not completely refined to white flour, the presence of polysaccharides may have a positive impact on hydration, rheological, and health-promoting properties. The tested flour used in the experiment was composed by Lewko et al. [41] from selected passages and is characterized by the high content of total AX (1.91%) and total NSP (3.40%) (Table 3).

We observed that all the applied treatments lowered T-AX content by decreasing insoluble and increasing soluble fractions of AX with more significant effect of cellulase addition than by combined cellulase–xylanase hydrolysis on AXs. A similar tendency was observed regarding T-NSP content. In both fractions, significant increase was seen for soluble AX and NSP, with the most significant effect being that of hybrid EFCX treatment on the increase of soluble components in the modified wheat flour. AXs are the major polymers in the cell wall of wheat grain, and therefore a major component of dietary fiber. In wheat, both soluble and insoluble fractions of polysaccharides are present, coming from the endosperm and outer layers [61]. AX fractions may be characterized by an arabinose-to-xylose ratio (A/X). Soluble fraction ratio of about 0.6 suggests more endosperm content in flour, whereas a ratio closer to 1 indicates that the polysaccharides come from the outer layers. We found in our F sample an I-A/X ratio at 0.723 and an S-A/X ratio at 0.842, indicating the presence of bran fractions in the tested flour. The composition of S-AX and I-AX (Table 3) in the developed flour F used in our study, with more insoluble fractions of AXs and NSPs [41], clearly showed that most insoluble fractions come from the outer layers than from endosperm (I-AX 1.31% and S-AX 0.60% in wheat flour F). Both enzymatic hydrolysis and extrusion and hybrid enzymatic–extrusion treatments lowered the amounts of insoluble fractions—mostly due to lowering of total AX content. The most significant decrease of I-AX and increase in S-AX amount were visible after hybrid extrusion of wheat flour with combined enzymes, but without changing T-AX. This outcome suggests that the EFCX treatment method has the greatest effect on the solubility of AX, in comparison to a slight effect brought about due to extrusion conditions.

Zhou et al. [36] used xylanase and alkali treatment to extract AXs from wheat bran, and they found A/X of 0.56 and 0.83, respectively, despite the molecular weight of alkali-treated AX being about 10 times that of xylanase-treated AX. Chen et al. [35] tested various methods of extraction of AX from triticale, and they found that the cellulase–xylanase extraction method followed by alkaline extraction from the residues of complex enzyme extraction to be the most suitable for obtaining the highest yield of AXs (with a 1.52 index of A/X, however, this ratio is only 0.25 A/X if pure enzymatic extraction was applied). Ma et al. [56] state that enzymatic methods with endoxylanases are efficient for extracting water-unextractable AXs and that a combined cellulase–endoxylanase extraction gives higher AX yields than xylanase, while the double-enzymatic extraction of AX from fresh corn fiber was more efficient than the chemical methods reported by other authors. Chen et al. [20] observed in wheat bran containing water and fed into the extrusion unit that the bonds of insoluble polysaccharides, for instance, cellulose and hemicellulose, and the continuous fiber matrix were partially disrupted and released soluble saccharides after extrusion due to the combination effects of high shear, turbulence, and cavitation, along with temperature. A similar observation was reported by Arcila et al. [58] as the extrusion with lower feed moisture (15%) was more effective in converting unextractable hemicellulosic fractions to extractable polysaccharides and increasing the amount of soluble dietary fiber components in extruded wheat bran due to more abrasion and mechanical disruption. This effect is opposite to the use of high moisture (30%) where water acts as a plasticizer in the extruder barrel. Moreover, the use of a corotating twin screw extruder at 50°C enhanced AX solubilization at low water content (below 54%), as compared to blade-mixing. Accordingly, extrusion enabled efficient enzyme action at low water content due to the enhanced diffusion of xylanase enzyme by the formation of a continuous mass in the extruder [62]. As found by Andersson et al. [63], the extractability of AX in wheat and rye bran increased depending on the extrusion parameters. They noted that the enhanced extractability of dietary fiber and AX, in combination with maintained content of *β*-glucan in wheat and rye bran, makes the extruded bran more valuable as additive in the food industry. Beyond the aforementioned, Fadel et al. [59] reported extrusion cooking to be an innovative pre-treatment useful to increase the solubility of AXs.

In Tables 4 and 5, a detailed composition of insoluble and soluble NSPs in native and modified wheat flour is presented. As seen in the tables, higher content of soluble fractions of NSPs was observed for mannose and galactose, but glucose, arabinose, and xylose higher content of insoluble fractions was observed, both in native and modified flour. The application of the hybrid method with combined enzymes (EFCX) showed strong decrease of I-arabinose and I-xylose (Table 4) and strong increase of soluble fractions of these polysaccharides (Table 5). When extrusion treatment was applied to wheat flour, all the results of S-A/X were lower than for unmodified F flour (Table 5), albeit, less important differences were noted for I-A/X ratio (Table 4). What is interesting, the addition of enzymes to wheat flour (FC and FCX samples) without other treatments increased the level of soluble fractions of all soluble polysaccharides (Table 5) as well as I-mannose, while other insoluble components were also lower after enzymatic action (Table 4). In our study, T-NSP content was significantly correlated with SDF and TDF, with correlation coefficients of 0.851 and 0.730, respectively. S-AX was also highly correlated with S-NSP (0.958) and I-NSP (−0.794) and with I-AX (−0.900), whereas for I-AX, slightly lower correlations were noted with S-NSP (−0.867) and I-NSP (0.827). Moreover, individual polysaccharide fractions showed correlations with protein content, for example, S-arabinose (0.706), S-xylose (0.678), S-AX (0.698), I-NSP (−0.662), and S-NSP (0.752). In addition, strong negative correlations were found between insoluble and soluble fractions of polysaccharides (arabinose, xylose, and AX with values from −0.801 to −0.910).

A strong correlation (0.98) was observed by Barron et al. [38] between the AX and TDF contents, indicating that AX can be used to estimate TDF content in wheat fractions and wheat-based food products. What is more, Kaur et al. [40] reported A/X ratios between 0.33 and 0.62 for brans of four different wheat varieties. These authors have found various ratios of A/X in bran fractions rich in AXs. They reported A/X ratios values for water-extractable fractions between 0.09 and 1.37, for alkali-extractable fractions values between 0.33 and 1.82, and for cellulosic AX values between 0.38 and 0.7. Hell et al. [64] used individual or enzyme mixes composed of cellulase from *Trichoderma reesei*, xylanase Pentopan Mono BG and GH 43 *α*-L-arabinofuranosidase from *Bifidobacterium adolescentis* to modify wheat bran after extrusion as one of the modification methods.

Demuth et al. [65] reported that application of twin screw extrusion at a maximum temperature of 133°C increased accessibility of cellulose, released more glucose, and observed high solubilization of xylose than in untreated wheat bran. Similar to our study, they reported a slight decrease in arabinose and xylose content after extrusion as compared to untreated wheat bran, which implies selectivity of this treatment towards AX. Demuth et al. [65] also speculated that after enzymatic extrusion the extrudates might be more accessible to xylanase, what can be valuable in preparation of feruloyl oligosaccharides (FOs). In addition, enzymatic extrusion might greatly shorten the time required to remove starch and protein as compared with traditional hydrolysis [2].

### 3.3. Rheological Characteristic of Native and Modified Wheat Flour

Rheological features, as tested with Mixolab device for untreated, extruded, and hybrid treated wheat flours, are presented in Table 6. According to the results of this work, hydration capacity was similar (around 60%) for F, even if enzymatic treatments (FC and FCX) were applied. A significant increase of Hyd was observed in all the extruded flours, and this varied from 94.3% for the EF27 sample, to 117.7% for EF23 and decreased significantly with higher feed moisture. Smaller differences were noted between hybrid enzymatic-extrusion treated samples regardless of the enzyme used. Dough development time (DT) in all tested modified flours (Table 6) was longer than indicated for F and the most significant differences were observed if cellulase (FC) and combined cellulase–xylanase (FCX) were added to wheat flour, in these cases, by 28% and 40% of time elongation, respectively. A connected parameter is a dough stability, with significant negative correlation to hydration capacity (−0.973) for all the samples. The application of enzymatic hydrolysis to wheat flour lowered only slightly the dough stability, whereas extrusion and hybrid enzymatic–extrusion treatments lowered dough stability by more than double, and for EF23 sample, even triple (Table 6). In EF- and EFC-treated flours, stability slightly elongated with increase of feed moisture, but if EFCX was applied, an opposite tendency was observed. The reduction in dough stability may therefore be related to the degradation of the gluten matrix occurring during the extrusion process due to the increase in temperature, as high-temperature treatments will modify the characteristics of the components of the gluten matrix [65].

Hyd capacity of extruded wheat flour rises with increasing treatment intensity, but dough stability tends to decrease [66]. The Hyd capacity of dough depends mainly on the flour composition and increases with increasing protein, pentosan, and damaged starch content. Indeed, the association between the quantity of damaged starch and the Hyd capacity of flour has been long established. Studies have also been performed with pregelatinized starches, which show a similar behavior to damaged starch because the starch granules break down during this process. Pasqualone et al. [67] reported increased Hyd after industrial scale extrusion cooking of lentil flour with two temperature/screw profiles (EF1: temp. 80°C–90°C–100°C, rpm 220, and EF2: temp. 100°C–105°C–115°C, rpm 230), they found a significantly higher Hyd for sample EF1 (94.7 g/100 g) extruded at lower temperature and screw speed than EF2 (90.8 g/100 g) and F (41.1 g/100 g).

Results of protein weakening (C2), starch gelatinization (C3), amylase activity (C4), and starch retrogradation (C5) of native wheat flour, enzyme hydrolyzed, extruded, and hybrid method treated are presented in Table 6. Significant differences were noted between samples that had passed through nonthermal and thermal modifications. Application of FC and FCX hydrolysis in environmental conditions caused only little effects on all rheological properties evaluated via Mixolab procedure. Increasing water addition to the extruded samples, however, brought about increasing values of C2, indicating more intensive protein weakening in the extruded samples without or with enzyme addition. This outcome can be an indicator of protein destruction after treatment. Starch gelatinization indicator (C3) showed the most significant differences in EF samples without enzymes extruded with various feed moisture: As water content increased, the C3 values raised. In addition, lower differences and opposite tendencies were found if enzymes were added during the extrusion. This outcome seems to indicate the effect of combined enzymatic–extrusion effect on starch gelatinization. The lowest values of C2, C3, C4, and C5 indicators were also found in EF23 samples characterized by the highest hydration, which might be the result of the most intensive treatment of wheat flour with limited water amount causing thermomechanical damage of both proteins and starchy components under twin-screw extrusion treatment (Table 6). The greatest differences (more than 2.5–3.0 times lower values) were noted for C4 and C5, indicating significant differences in amylase activity and starch retrogradation of the extruded flour, as compared to native and enzyme that were only hydrolyzed. The effect of hybrid cellulase-extruded and combined cellulase–xylanase extrusion was negligible without specific effects of enzyme addition or water dosing on rheological characteristics measured with Mixolab. Statistical analysis showed that C3, C4, and C5 results were in strong negative correlation with hydration of the tested flours (−0.974, −0.963, and −0.965, respectively). Moreover, high and significant correlations were found between stability of dough and C3 (0.994), C4 (0.996), and C5 (0.995) of tested modified wheat flour. Unlike the total AX, which contributes towards good viscoelastic properties of wheat flour dough, the A/X ratio does not have any remarkable effect on the rheological properties of wheat flour dough, as reported by Kaur et al. [40]. Rheological properties of raw materials and pasting or hydration characteristics explain the technofunctional properties of wheat flour and dough. Some authors reported that the addition of AX influenced rheological and mixing properties of dough and Hyd due to high water binding capacity [37]. During the bread-making process, addition of AXs can be advantageous due to their high water binding capacity [40].

### 3.4. Pasting Properties of Native and Modified Wheat Flour

Pasting properties indicate the thermal sensitivity and ability of starches or flours to create a viscous structure under heating and cooling and can support to identify the retrogradation tendency by breakdown and setback results. The results of pasting properties of native, enzyme-treated, extruded, and hybrid-treated wheat flours are presented in Table 7. Native wheat flour tested under Viscograph procedure showed max viscosity of 745 BU (which corresponds to 1564.5 mPa·s), application of cellulase FC, and FCX lowered max viscosity significantly to 625 and 573 BU, respectively. This suggests the partial hydrolysis of polysaccharides due to applied enzyme activity by degrading insoluble cellulose to soluble sugars by cellulase and hydrolyzing xylans (e.g., AXs and glucuronoxylans) by xylanase.

**Figure 1:**
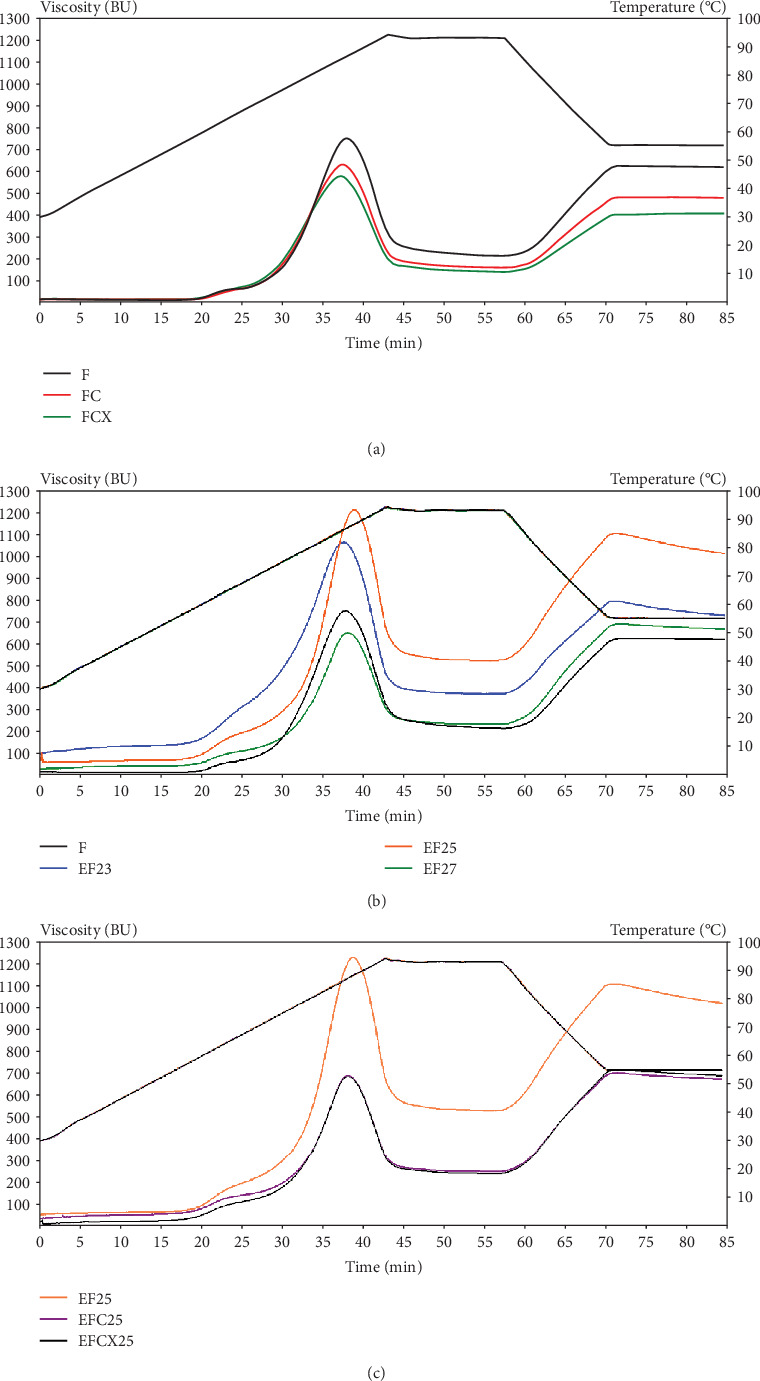
Pasting curves of wheat flour modified under various conditions: (a) native wheat flour, with cellulase (FC) and cellulase–xylanase (FCX), (b) extruded at various feed moisture without enzymes, and (c) hybrid extruded at 25% moisture with cellulase (EFC25) and cellulase–xylanase (EFCX25).

The combined effect of both enzymes acting together demonstrated more significance on pasting properties, both in F and in hybrid enzymatic–extrusion treatment. Pasting properties of flour samples processed with addition of enzymatic complex EFCX showed significant lowering of maximum viscosity, trough viscosity, and final viscosity values, as well as breakdown and setback as compared to samples extruded with addition of cellulase or extruded without enzymes. Moreover, this trend was strongly visible if a higher level of initial moisture was applied during processing (EFCX27) as compared to the only extrusion treatment (EF27). Thus, the application of enzyme addition, especially CX complex, to extrusion process performed at the highest initial moisture level, allowed to obtain the lowest maximum viscosity (546 BU), trough viscosity (174 BU), and final viscosity (520 BU) as well as breakdown (372 BU) and setback (346 BU) from among the extruded samples, so CX enzymes lowered the effect of extrusion treatment at high moisture. Differences in pasting behavior are presented in Figure 1 depending on treatment methods. The application of low-temperature extrusion cooking of native flour (EF) under various feed moistening caused increase of max viscosity if 23% and 25% feed moisture was applied. At the highest moisture (27%), applied max viscosity was even lower even that for native F, but higher than observed for flour modified by enzymes only (FC and FCX). Hybrid enzyme–extrusion treatment showed slightly higher viscosity if extruded at 23% moisture due to the more intensive mechanical treatment at low moisture; other conditions, however, did not significantly affect wheat flour viscosity; the results obtained were similar to that for pure enzyme hydrolysis [17]. Trough viscosity or holding strength means the trough at the minimum hot paste viscosity and could be connected with water holding capacity of swollen starch after heating and cooling stages [68]. Trough viscosity decreased after enzyme addition but was higher than in F when extrusion treatment was applied, especially at 23% and 25% of feed moisture. Significant tendency to decrease the final viscosity with increasing flour feed moisture was noted for both enzyme type applied, but the results for hybrid enzymatic–extrusion treatment were higher by around 30% than that for only enzymatically hydrolyzed wheat flour (FC and FCX). The application of individual enzymes significantly lowered breakdown and setback values due to changes in various fractions of polysaccharides via partial hydrolysis of carbohydrates by cellulose and combined cellulase–xylanase action. We also noted that breakdown and setback values were significantly correlated with max viscosity (0.961 and 0.733, respectively). The temperature of beginning gelatinization was similar (60.2°C–60.5°C) for all tested native flours without (F) or with enzymes (FC and FCX), so any effect of enzymes on gelatinization tendency was notable. In the extruded samples treated by thermal and mechanical treatment, as well as by hybrid treatment at temperatures up to 80°C, partial gelatinization occurred inside the extruder; hence, the temperature registered for the beginning of gelatinization were from 35.2°C to 42.4°C when 25% and 27% feed moisture was applied. Lower gelatinization temperature was reported in the EF23 sample.

This effect was due to more intensive treatment under low moisture content, which results in greater starch degradation due to intensive mechanical shearing. Moreover, great correlation was found between setback and fat content (0.974), the extruded samples were much lower in fat, probably due to formation of lipid–starch or lipid–protein complexes through the extrusion processing [1], and this affected higher setback in these samples.

Robin et al. [17] reported a peak viscosity of the raw whole wheat flour at 1800 mPa s via RVA pasting profile, and after extrusion, peak viscosity lowered significantly (from 117 to 292 mPa s), suggesting that extrusion disrupted the crystalline organization of starch, generating an amorphous state. However, full gelatinization of the starch granules did not occur because of limited water content during extrusion (ranging between 18% and 22%), and increasing the water content led to a decreased degree of starch transformation as indicated by the highest viscosity being at maximum water content and minimum screw speed. De Pilli et al. [31] studied the effects of two commercially available enzymes (protease and amylase) on selected properties of products obtained from wheat and almond flour extruded at low temperature (54°C) via a twin-screw extruder. They reported a decreased dough viscosity during the extrusion at increased moisture content due to the pressure decreasing at high dough moisture. This trend could be attributed to a reduction of enzymatic activity caused by elevated moisture content. Water is essential to provide the catalytically active conformation of the enzymatic system. However, during the denaturation process, water behaves as a plasticizer, which allows the enzyme molecules to unfold, causing them to lose their native conformation. Mitrus et al. [12] observed that the addition of increased amounts of water raised the peak viscosity of extruded bean flour, and the results showed that the twin-screw extrusion cooking process reduced the retrogradation tendency of bean paste due to starch degradation occurring during processing because of value breakdown decrease and setback increase with increased water feeding. Pasqualone et al. [67] tested extruded lentil flour and reported increase of viscoamylograph initial viscosity compared with F, with significantly higher value when lower temperature and lower screws speed were applied. Additionally, they noted lower degree of starch retrogradation in extruded lentil flours than in native one. Román et al. [29] studied the pasting profile of wheat flours, and they reported lower viscosity profiles of nonenzymatically treated and extruded flours, similarly as in our research, what confirmed the gelatinization process occurring during thermal treatments. Values of breakdown and setback were also reduced. Tests performed on both extruded and native samples with additional enzymatic treatment revealed flat pasting profile curve with absence of characteristic peak viscosity and very low viscosity values as a result of the enzyme hydrolytic activity on the starch [29]. Uthumporn et al. [33] found in heat-treated corn and potato starch significant increase in setback viscosity compared to control starches, indicating great tendency to retrogradation. After enzymatic hydrolysis, where amylose was degraded into shorter chain oligosaccharides that are easier to reassociate and solubilize, the setback values increased, showing positive correlation with the solubility. Deng et al. [2], in turn, tested rice bran processed via the enzymatic–extrusion method and found higher viscosity of bran extruded at low screw rate and at low moisture content due to the higher specific mechanical energy input from the extruder at limited access to water, which softened the fiber. The higher mechanical energy input is most likely caused by the formation of a loose and porous structure that facilitates the penetration of the xylanase-containing solution.

### 3.5. Hydration Characteristics of Native and Modified Wheat Flour

SRC may be a suitable indicator of the true hydration ability of flour. In contrast, flour is subjected to mechanical forces or kinetic effect during the measuring of Hyd by Mixolab or RVA, and the true value may be effected by increased starch or protein mechanical damage [50]. The SRC method also enables identifying separate functional contributions from damaged starch (Sc solvent), pentosans (Su solvent), and gluten (La solvent) at the same time—mostly identifying differences during flour milling intensity. Flour dedicated to various applications are characterized by holding specific properties, for example, for bread production, high Hyd, good gluten strength, and relatively high damaged starch and AXs are required, but for cookies, minimal gluten strength, low damaged starch and AXs, and low Hyd are recommended.  Table 8 shows the SRC values of untreated, extruded, and hybrid-treated wheat flours tested via various media. In our work, F (nontreated) was characterized by low SRC to all tested solvents as compared to the extruded. Moreover, cellulase and combined cellulase–xylanase hydrolysis of F only slightly increased SRC values, in many cases without significant differences between F and FC or FCX samples. Application of extrusion increased more than triple SCRWa, more than double SRCSu and SRCLa, and almost four times SRCSc of the extruded flour at the lowest feed moisture. With increasing water addition during simple extrusion of wheat flour without enzymes, a significant and constant lowering of all SRC results was observed. In hybrid enzymatic–extrusion-treated samples, the range of SRC values showed similar tendency if cellulase was used individually (EFC samples), whereas in EFCX hybrid-treated samples, the results were not linearly dependent on water addition and the highest SRC values were observed if 27% of moisture was applied in relation to SRC of Wa, La, and Sc solvents.

Water-soluble pentosans (also known as water-extractable AXs) are considered to have great water holding capacity, and this property may explain increased hydration of solvents due to increasing content of soluble fractions of AXs (Table 2) in the tested flours modified with extrusion (EF) or hybrid treatment (EFC and EFCX), as well as because of damaged starch or gluten by extrusion treatment—as confirmed by significantly lower gelatinization temperatures obtained in pasting tests. GPI can calculated based on the SRC results. GPI has been found to be an even better predictor of the overall performance of flour glutenin in the environment of other modulating networks of flour polymers, for example, in bread [50]. Results presented in Table 8 for GPI of native, enzymatic-treated, and hybrid-treated flour clearly indicated significant decrease of GPI in extruded samples without significant effects of the enzymes used in hybrid treatments. GPI values were strongly negatively correlated with SRC Wa, Su, La, and Sc (−0.957, −0.928, −0.923, and −0.964, respectively). All SRC and GPI results were, surprisingly, significantly positively correlated with fat content in the tested flours (correlation coefficients from 0.903 to 0.981) and with Hyd (from 0.989 to 0.994), whereas a strong negative correlation was found between Hyd and GPI (−0.961). Moreover, significant positive relations of setback (from 0.650 to 0.711) and negative of gelatinization beginning temperature (from −0.916 to −0.965) were found with all SRC results. The results of the work of Moreno-Rivas et al. [55] showed that extruded nixtamalized corn flour, with and without xylanase, had increased protein solubility, compared to nontreated and nonextruded, and this effect was lower in extruded nixtamalized corn flour with xylanase. Moreover, insoluble protein diminished in corn flours either with or without xylanase enzyme. The addition of xylanase reduces the effect that the extrusion process has on the solubility proteins of extruded nixtamalized corn flour.

### 3.6. Structure Diffraction Analysis of Modified Flours

**Figure 2:**
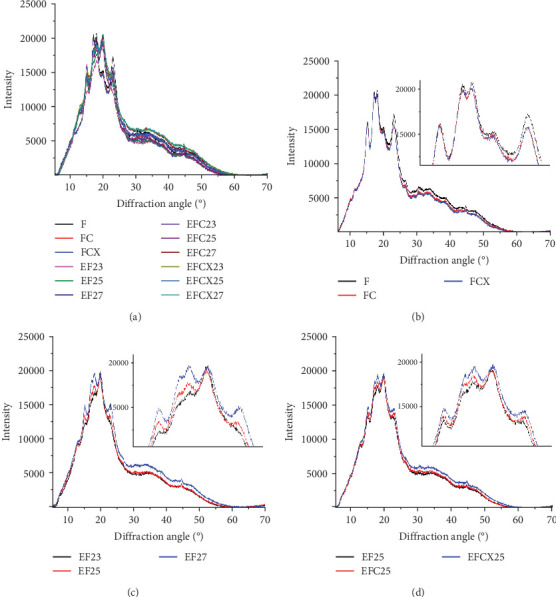
X-ray diffraction patterns of native, extruded and hybrid enzymatic–extrusion-treated wheat flour: (a) comparison of patterns of native (F) and extruded (EF) samples without or with enzymes, (b) effect of enzymes addition to native wheat flour, (c) effect of feed moisture of extruded wheat flour without enzymes, and (d) effect of enzymes addition extruded samples at 25% feed moisture. C: cellulase; CX: cellulase–xylanase complex.

To analyze the internal structure of native and extruded wheat flour without or with enzyme addition processed at variable moisture levels, we applied the XRD technique. The XRD patterns identified in native and extruded and hybrid treated wheat flour samples are collected in Figure 2a. Two main profiles were identified in wheat flour: for native and enzyme hydrolyzed flour (Figure 2b) and for extruded and hybrid enzymatic–extruded flour (Figure 2c,d). The Fs, rich in starch, with present A (large) and B (small) granules of wheat starch displayed typical A-type XRD patterns at 2*θ* with sharp peaks: a first peak around 15°, a strong doublet near 17° and 18°, and a third main reflection around 23°. These are characteristic for the crystalline structures of cereal starches. In native samples (F, FC, and FCX), only a small peak at 20° was noted. Such effect (2*θ* ≈20°) represents the amorphous peak of amylose and lipids, and these are in low amounts in native wheat flour, and, indeed, were found to decrease slightly more after enzymes addition (Figure 2b). Other enzymes effects were visible in FC and FCX samples at 23° as compared to F. Such results indicate that native wheat kernel starch had typical A-type crystal characteristics [69, 70]. After the extrusion, much higher peak intensity at 2*θ* ≈20° was observed, both without (Figure 2c) or with enzymes addition (Figure 2d)—indicating higher content of amylose and lipids complexes in the extruded flour. This outcome is confirmed by the lower extractable fat content in this (Table 2). Moreover, changes in peak intensity at 2*θ* ≈23° were observed. In the extruded samples, these peaks were lower in intensity than that for the Fs. Lower relative intensity of peaks at 2*θ* ≈15° were also seen, especially when low feed moisture 23% was used during extrusion (Figure 2c). With regard to the effect of moisture content, a diminished peak at 18° was found with limited water amount (EF23), with absence of a 17° angle peak (almost absent in EF25, but visible in EF27). If high feed moisture was applied during processing, a larger surface area under the curve was observed in the region between 25° and 50°, suggesting higher amounts of structure with amorphous characteristics after extrusion. Some changes in this region were also observed with enzyme addition: if CX complex was used, the intensity of 15° peak was higher (Figure 2d) and the surface area under the curve between 25° and 50° was greater when complex CX was added (as seen in the EFCX25 and EFCX27 samples) (Figure 2a,d). The strong doublet peaks around 2*θ* ≈17° and 18° were not observed in the extruded wheat flour, suggesting a transformation of starch crystalline structure in this region. Similar observations were done by Lewko et al. [71]. After the extrusion treatment (without or with enzymes), significant changes were observed at the 20° diffraction angle. In this region, the F showed a low intensity peak (Figure 2b), whereas after extrusion, in all extruded samples, this peak was very intensive with the altitude similar in intensity as for 18° (Figure 2c,d). As stated previously, Liu et al. [69] identified the peak at 2*θ* ≈20° as the amorphous peak of amylose and the lipids. A higher peak intensity indicates a higher content of amylose and lipid complexes formed after extrusion. This effect was confirmed by the notable lowering of extractable fat content, as compared to native (nonextruded) flour. Additionally, microstructure analysis confirmed the formation of an amorphous melted structure after extrusion, with singular starch granules embedded in a continuous starch–protein matrix, as confirmed by SEM. Zeng et al. [70] found XRD patterns to be true “fingerprints” of the crystal structure within starch grains. Accordingly, the crystal structure of starch can be identified as one of four types (A, B, C, and V types) due to the presence of characteristic XRD lines at proper diffraction angles. A, B, and C types indicate the crystalline structures commonly present in natural cereal starches, and, while V type is crystalline, it is typical of the complexes formed by amylose and lipids. In our study, the A-granules with larger particle size show sharper XRD patterns than the other starches, and this may be the explanation for the flattening of the curve of diffraction due to the structure differences in native and extruded samples as observed on SEM microscopic pictures, where after extrusion, only a few swollen granules are present.

**Figure 3:**
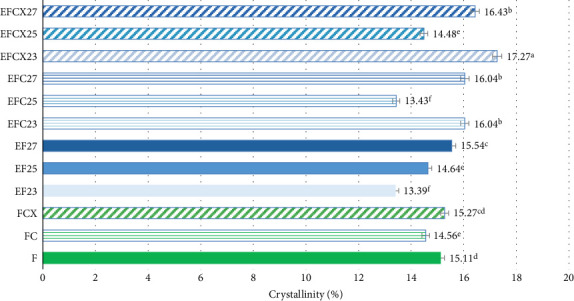
Crystallinity results of untreated, extruded, and hybrid-treated wheat flours.

Crystallinity of the tested samples as Fs without or with enzymes as well as extruded and hybrid enzymatic–extruded flours was obtained on the base of XRD results obtained at the range of *θ*–2*θ* from 5° to 70° (4922 points) fitted to a Gauss–Cauchy model. Two models were used for calculations due to significant changes between native and extruded XRD curves and peak number and intensity. Fs without and with addition of enzymes were analyzed as a function of 15 peaks. In the extruded flours the curves obtained were less sharp with 13 significant peaks taken for fitting the model. The results are presented in Figure 3. Native wheat flour was characterized 15.11% of crystallinity and addition of enzymes did not affect significantly on crystallinity changes in nonprocessed flours. For a native wheat, Saiah et al. [72] and Leblanc et al. [73] confirmed the presence of an A-type structure, characteristic to cereal starches. They noted after measurement at *θ*–2*θ* from 5° to 35° a degree of crystallinity at 40% *w*/*w* for wheat starch and at 30% *w*/*w* for the wheat flour because of the presence of highly amorphous multipolymer gluten in wheat. Yoo and Jane [54] tested structural properties at the range of *θ*–2*θ* from 4° to 40° of starches isolated from waxy wheat, amylose-reduced Kanto 107 variety, normal hard red winter wheat Centura, and commercial wheat starch and they noted 18.0, 14.5, 12.0, and 13.0% percentage crystallinity calculated based on X-ray diffractograms. Waxy wheat starch had significantly greater crystallinity than others.

Differences in crystallinity of native and extruded samples were small, probably because the A-type crystallinity became rearranged and V-type is formed in the extruded samples containing starch due to its melting under treatment via extrusion [72–74]. The crystallinity in extrudates is also the effect of formation of complexes between amylose and endogenous lipids (amylose–lipid complex). Amylose–lipid complexes are generally produced after gelatinization of starch in a presence of heat and water due to starch melting during extrusion cooking and destruction of amylopectin double helices, whereas part of the free lipids can form a helical inclusion complexes with the amylose molecules [75]. This confirms also the low fat content in our extruded wheat flour samples, as presented in Table 1, and small differences in crystallinity due to the calculation method based on the ratio of area under the curve of crystalline and amorphous structures. The most significant effect of lowering crystallinity (13.39%) was observed in wheat samples extruded alone at the lowest feed moisture, as visible in Figure 2c, and extruded with cellulase or xylanase–cellulase complex at 25% of feed moisture (13.43 and 14.48%, respectively). Increase in initial moisture when wheat flour was extruded caused increase of crystallinity to 15.54% when 27% feed moisture was applied. This may be the effect of greater starch gelatinization at increased access to water under 80°C–85°C extrusion temperature and more intensive changes from A-type structure into V-type (both crystalline) what was observed also on diffraction curve (Figure 2c) as a highest peak at 23°. Oliveira et al. [74] reported that the loss of crystallinity in extruded products is the effect of mechanical breaking of molecular bonds as a result of intense shear forces inside the extruder. Therefore, in the case of low-moisture extrusion, typical for expanded products, small amounts of starch in the gelatinized and melted state are mixed and simultaneously fragmented. They found values of crystallinity of corn flour and wholegrain wheat flour in the range of 16.97%–23.81% for the extrudates treated in L/D 29 twin screw extruder at 75°C and 100°C and 16%–22% feed moisture against 25.20%–34.00% for unprocessed flours and flour blends, but samples were tested within a narrower range of *θ*–2*θ* from 3° to 35° at a scanning rate of 1°/min. They noted no significant effect of any variable studied on extrudate crystallinity despite differences in crystallinity values. In our research the highest crystallinity (17.27% and 16.43%) was observed in flour with xylanase-cellulase complex extruded both at 23% and 27% of initial moisture, respectively, and similar tendency was noted if the only cellulase was added before extrusion. Application of 25% feed moisture in both cases (EFC25 and EFCX25) limited formation of crystalline phase when wheat flour was supported by enzymes before extrusion. Jafari et al. [75] tested native sorghum flour, exhibiting A-type XRD patterns with crystallinity of 30.84%, as well as extruded sorghum flour. Extrusion cooking lowered crystallinity in the range of 17.59%–29.94% depend on processing conditions when sorghum flour was extruded at 110°C and 160°C with 10%, 14%, and 18% of food moisture in a corotating twin screw extruder with L/D 10. A-type and V-type crystalline peaks became narrower when feed moisture and die temperature decreased, what suggest reorganization of helices into packed structures but they tested only range of 4°–40° so any amorphous structures over this range were not taken into account, as in our case. Increasing shear forces intensity at low moisture content could disrupt molecular bonds increasing the availability of amylose chains while the high temperature could promote molecular movement and more amylose-lipid complexes could be formed at low moisture content and high temperature during extrusion process. So, decreasing of feed moisture and increasing of die temperature showed a negative effect on starch crystallinity in extruded samples [74]. Similar effect was observed in our extruded wheat flour (samples EF23, EF25, and EF27, Figure 3). After a single screw extrusion at 120°C and 40 rpm Saiah et al. [72] and Leblanc et al. [73] found peak located at 22.6° characteristic for a V-type crystalline structure, but also the peak located at 17.3° of very small magnitude indicating the existence of a residual A-type crystalline structure in the extruded materials with the degree of crystallinity of 14% in all extruded samples. Similar peaks (2*θ* ≈17° and 23°) were observed in our native and extruded wheat flour samples with various intensity (Figure 2). A very small fraction of granules (observed on microscopic pictures) may be not transformed in the extrusion process due to incomplete restructuring under presented conditions.

### 3.7. Microstructure Observations

**Figure 4:**
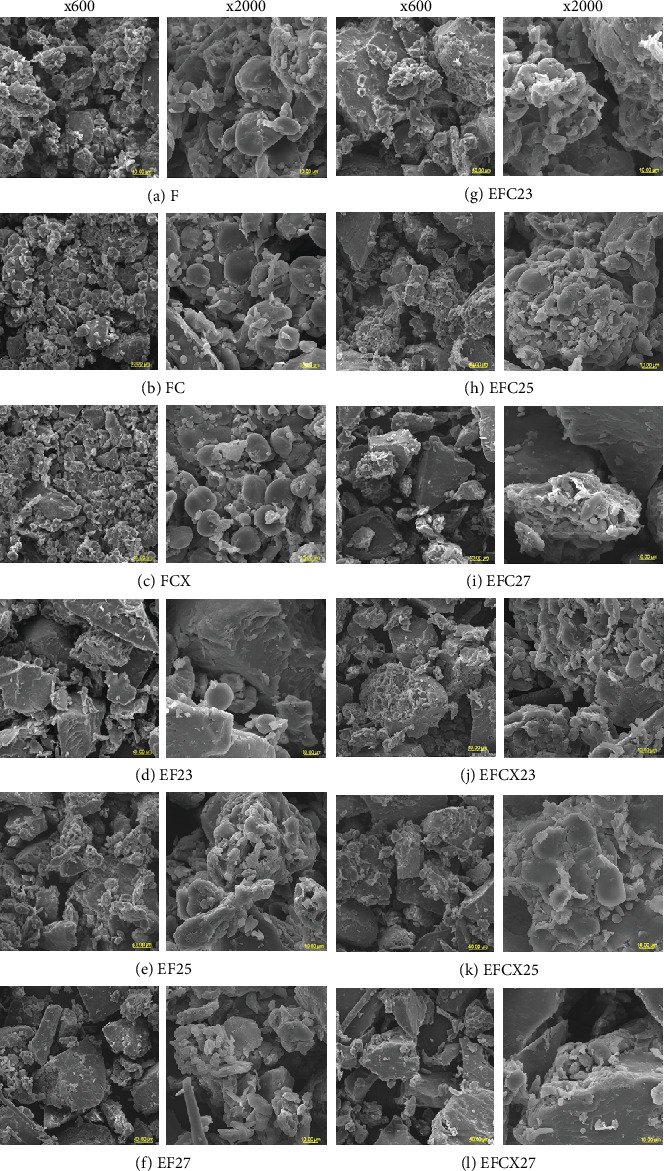
SEM micrographs of flours at low (×600) and high (×2000) magnifications. Nontreated (a), enzymatically treated with C and CX (b, c), extrusion-treated (d–f), hybrid enzymatic–extrusion treated with C (g–i) and CX (j, k, l) flours processed at 23%, 25%, and 27% of feed moisture, respectively.

Microstructure observations with SEM presented in Figure 4 indicated that the addition of enzymes had only a minor impact on the internal structures of FC and FCX flours (Figure 4b,c), as compared to native (Figure 4a). Moreover, in samples with both enzymes, the partly swollen starch granules were visible as larger amounts of finer granules (Figure 4b,c) when compared with F (Figure 4a). The A-starch granules also displayed a disk shape with diameters of 12–28 *μ*m, and the isolated B- starch granules appeared as a spherical shape with diameters of about 2.5–8.5 *μ*m, while very minute (< 2.0 *μ*m) C-type starch granules were indicated—although this type of granule may represent a B-type granule [70]. We also observed some fibrous fractions coming from the outer layer of the grains used for the developed flour composition. This suggests the presence of polysaccharides in native wheat flours. Similar observations were made by Zeng et al. [70] for white and wholegrain wheat flour. Here, they observed two types of starch granule distribution: large granules measuring about 35 *μ*m and small granules about 2–10 *μ*m. Application of low-temperature extrusion processing caused significant changes in the structure of the modified flours. In all extruded (EF) and hybrid-extruded (EFC and EFCX) samples, melting and agglomeration of components were observed, with only singular starch and fiber particles visible (under high magnifications). In Figures 4d, 4e, 4f, 4g, 4h, 4i, 4j, 4k, and 4l, for example, swollen starch granules embedded in the amorphous structure of gelatinized starch-protein matrix are visible in a molten mass under various magnifications (×600 and ×2000). The extruded wheat flour also presented almost null presence of native starch granules with a defined structure. Similar findings were reported by Lewko et al. [71]. The effect of increasing feed moisture on more compact and homogenous internal structure was observed especially in samples extruded at 27% of moisture both without (Figure 4f) and with enzyme addition (Figure 4i,l), where a more uniform melted phase was noted than in samples with lower moisture during processing without or with enzymes (Figures 4e, 4h, and 4k, respectively). In samples processed at 23% moisture, starch ungelatinized granules were observed (Figures 4d, 4g, and 4j). This suggests that insufficient water was available for starch to complete gelatinization at the temperature range used in the experiment. The application of cellulase and xylanase-cellulase complex created a loose structure of melted fractions with visible particles inside agglomerates formed by the extrusion process, especially at low feed moisture (Figure 4g,j).

Other authors observed changes in the microstructure of modified flours or starch after extrusion processing. Moreno-Rivas et al. [55] reported remarkable damage and surface erosion of starch granules after extrusion, although such structure changes were also observed in conventional nixtamalization as an effect of lime presence and soaking time. Liu et al. [76] reported significant changes in microstructure of rice starch after extrusion at 30%–70% of moisture, with almost complete gelatinization and shape destruction of starch treated with the highest moisture. Enríquez-Castro et al. [77] observed significant reduction in the number of granules and an increase in the irregular shape and pore surface in the microstructures of extruded nixtamalized corn flour. This effect probably came about due to higher enzymatic hydrolysis and starch digestibility. Extrusion cooking carried out with low moisture contents causes a high amount of starch granules to be fragmented and embedded in the endosperm matrix, as well as for some granules to be dispersed out of it. Under thermal treatment, especially extrusion, agglomeration of starch granules results in amorphous structures, and dextrinization is possible. Alam et al. [78], for example, applied twin screw extruder at temperature up to 130°C, but with limited feed moisture (17% and 19%) to obtain crispy products with high content of fiber coming from rice bran. Jafari et al. [75] observed microstructure of native and extruded sorghum flour and they found significant variation in starch granule shapes. Moreover, for mildest extrusion condition (10% feed moisture at 110°C) some nongelatinized starch granules were observed. However, with more intensive extrusion condition most of starch granules were completely melted and visible as agglomerated particles, similarly to our observations.

## 4. Conclusions

The obtained results confirmed that hybrid enzymatic–extrusion modification has a greater affect upon polysaccharide composition and the technofunctional properties of wheat flour than individual enzymatic treatment or extrusion processing. In our work, a specific effect of hybrid enzymatic-extrusion treatment of wheat flour was observed on polysaccharides fractions, the outcome of which significantly decreased insoluble AX content and increased soluble AXs and NSP content if a combined cellulase-xylanase blend was used during extrusion (EFCX samples). Moreover, all treatments involving low-temperature extrusion (without or with enzymes) caused lowering of fat content in the modified flours due to the formation of amylose-lipids complexes confirmed by crystallinity analysis. In addition, extrusion treatments at lower feed moisture without or with enzymes increased viscosity, breakdown, hydration, C2 and all solvents retention capacity, but decreased setback, stability, C3, C4, C5, and gluten performance index of the modified wheat flour. In addition, combined cellulase–xylanase hydrolysis showed a stronger effect on wheat flour modification than did cellulase treatment alone, especially by lowering viscosity and pasting properties and SCR values, and by increasing soluble AX and NSP, hydration, dough DT, and GPI in the modified flour. Hybrid enzymatic–extrusion modified wheat flour treated at proper processing conditions may be used in the bakery industry as a source of soluble AXs, as a thickening agent, or as a binder with high Hyd and solvent retention capacity and lower setback (which indicates the decreasing retrogradation tendency of the modified flour). The presented results can be helpful in the processing of wheat flour with specific technofunctional properties. Moreover, the practices enable this flour to be listed as a “clean label” additive. Application of modified flours as bread improvers will be tested in the next research step.

## Figures and Tables

**Table 1:** Experiment design and variables coded.

**Sample**	**Treatment**	**Enzyme type**	**Enzyme content (ppm)**	**Moistening level (%)**
F	Native	—	—	—
FC	Native	Cellulase	120	—
FCX	Native	Cellulase+xylanase	60 + 50	—
EF23	Extruded	—	—	23
EF25	Extruded	—	—	25
EF27	Extruded	—	—	27
EFC23	Extruded	Cellulase	120	23
EFC25	Extruded	Cellulase	120	25
EFC27	Extruded	Cellulase	120	27
EFCX23	Extruded	Cellulase+xylanase	60 + 50	23
EFCX25	Extruded	Cellulase+xylanase	60 + 50	25
EFCX27	Extruded	Cellulase+xylanase	60 + 50	27

Abbreviations: C: cellulase enzyme, E: extrusion treatment, F: flour, X: xylanase enzyme.

**Table 2:** Proximate chemical composition of untreated, extruded, and hybrid-treated wheat flours.

**Sample**	**Moisture content (%)**	**Protein (%)**	**Fat (%)**	**Ash (%)**	**IDF (%)**	**SDF (%)**	**TDF (%)**
F	13.90 ± 0.10^a^	14.62 ± 0.06^e^	1.31 ± 0.0^b^	0.72 ± 0.02^f,g^	3.94 ± 0.04^b^	2.86 ± 0.02^b^	6.80 ± 0.03^c^
FC	13.86 ± 0.09^a^	14.52 ± 0.03^e^	1.39 ± 0.02^a^	0.74 ± 0.02^d,e,f^	3.81 ± 0.03^c^	2.33 ± 0.02^e^	6.14 ± 0.05^d^
FCX	13.76 ± 0.07^a^	14.64 ± 0.01^e^	1.30 ± 0.01^b^	0.73 ± 0.01^e,f,g^	4.10 ± 0.03^a^	2.56 ± 0.03^c^	6.66 ± 0.06^c^
EF23	10.32 ± 0.12^b,c^	14.66 ± 0.03^d,e^	0.16 ± 0.02^e^	0.76 ± 0.01^d,e,f^	3.08 ± 0.03^f^	2.42 ± 0.02^d^	5.50 ± 0.03^g^
EF25	10.54 ± 0.10^b^	14.80 ± 0.03 ^b^	0.29 ± 0.02^c^	0.82 ± 0.02^a,b,c^	3.79 ± 0.03^c^	1.83 ± 0.02^h^	5.53 ± 0.15^g^
EF27	10.42 ± 0.08^c^	14.76 ± 0.02^b^	0.21 ± 0.01^d^	0.77 ± 0.02^d,e^	3.06 ± 0.03^f^	2.24 ± 0.02^f^	5.30 ± 0.02^h^
EFC23	10.30 ± 0.12^b,c^	14.67 ± 0.02^c,d,e^	0.23 ± 0.01^d^	0.78 ± 0.02^b,c,d^	3.37 ± 0.03^e^	2.57 ± 0.02^c^	5.94 ± 0.01^e^
EFC25	10.39 ± 0.10^b,c^	14.17 ± 0.03^g^	0.20 ± 0.02^d,e^	0.85 ± 0.02^a^	3.43 ± 0.02^e^	2.08 ± 0.02^g^	5.50 ± 0.03^g^
EFC27	10.22 ± 0.09^b,c^	14.66 ± 0.03^d,e^	0.16 ± 0.02^e^	0.77 ± 0.02^d,e^	4.18 ± 0.03^a^	2.93 ± 0.03^b^	7.12 ± 0.02^b^
EFCX23	10.22 ± 0.07^c^	14.73 ± 0.03^b,c,d^	0.29 ± 0.03^c^	0.78 ± 0.02^c,d^	3.62 ± 0.03^d^	4.12 ± 0.03^a^	7.74 ± 0.04^a^
EFCX25	10.21 ± 0.20^c^	14.76 ± 0.03^b,c^	0.22 ± 0.02^d^	0.70 ± 0.02^g^	2.91 ± 0.04^g^	2.61 ± 0.03^c^	5.52 ± 0.01^g^
EFCX27	10.31 ± 0.30^b,c^	14.90 ± 0.02^a^	0.30 ± 0.03^c^	0.82 ± 0.02^a,b^	3.13 ± 0.03^f^	2.56 ± 0.05^c^	5.70 ± 0.02^f^

*Note*: *n* = 3.

Abbreviations: C: cellulase enzyme; E: extrusion treatment; F: flour; IDF: insoluble dietary fiber; SDF: soluble dietary fiber; TDF: total dietary fiber; X: xylanase enzyme.

^a–g^Means indicated with similar letters in columns do not differ significantly at *α* = 0.05.

**Table 3:** Nonstarch polysaccharides and arabinoxylan content in untreated, extruded, and hybrid-treated wheat flours.

**Sample**	**T-AX (%)**	**I-AX (%)**	**S-AX (%)**	**T-NSP (%)**	**I-NSP (%)**	**S-NSP (%)**
F	1.91 ± 0.06^a^	1.31 ± 0.04^a,b^	0.60 ± 0.02^e^	3.40 ± 0.00^b^	2.06 ± 0.01^a,b^	1.34 ± 0.00^c,d^
FC	1.75 ± 0.02^a,b^	1.13 ± 0.02^c^	0.62 ± 0.01^e^	3.20 ± 0.04^c,d^	1.87 ± 0.03^d,e^	1.33 ± 0.01^c,d^
FCX	1.81 ± 0.00^a,b^	1.17 ± 0.01^a,b,c^	0.64 ± 0.01^e,d^	3.34 ± 0.01^b,c,d^	1.92 ± 0.01^c,d,e^	1.42 ± 0.00^b,c^
EF23	1.76 ± 0.06^a,b^	1.12 ± 0.05^c^	0.64 ± 0.01^e,d^	3.27 ± 0.05^b,c,d^	1.87 ± 0.00^d,e^	1.40 ± 0.05^b,c^
EF25	1.75 ± 0.07^a,b^	1.10 ± 0.02^c^	0.65 ± 0.05^e,d^	3.24 ± 0.04^c,d^	1.85 ± 0.05^e^	1.39 ± 0.01^b,c,d^
EF27	1.81 ± 0.04^a,b^	1.20 ± 0.02^a,b,c^	0.61 ± 0.02^e^	3.27 ± 0.03^b,c,d^	1.97 ± 0.01^b,c,d^	1.31 ± 0.02^d^
EFC23	1.74 ± 0.06^a,b^	1.14 ± 0.02^c^	0.59 ± 0.03^e^	3.35 ± 0.07^b,c^	2.02 ± 0.02^a,b,c^	1.33 ± 0.06^c,d^
EFC25	1.75 ± 0.06^a,b^	1.31 ± 0.06^a^	0.44 ± 0.00^f^	3.18 ± 0.04^d^	2.14 ± 0.03^a^	1.04 ± 0.01^e^
EFC27	1.84 ± 0.13^a,b^	1.16 ± 0.12^b,c^	0.68 ± 0.01^d^	3.30 ± 0.06^b,c,d^	1.83 ± 0.04^e^	1.47 ± 0.02^b^
EFCX23	1.82 ± 0.00^a,b^	0.88 ± 0.02^d^	0.94 ± 0.01^b^	3.64 ± 0.06^a^	1.91 ± 0.01^c,d,e^	1.73 ± 0.05^a^
EFCX25	1.73 ± 0.04^b^	0.85 ± 0.04^d^	0.88 ± 0.02^c^	3.29 ± 0.04^b,c,d^	1.63 ± 0.02^f^	1.66 ± 0.06^a^
EFCX27	1.84 ± 0.05^a,b^	0.83 ± 0.07^d^	1.01 ± 0.02^a^	3.25 ± 0.12^b,c,d^	1.54 ± 0.11^f^	1.70 ± 0.01^a^

*Note*: *n* = 3.

Abbreviations: C: cellulase enzyme; E: extrusion treatment; F: flour; I-AX: insoluble arabinoxylans; I-NS: insoluble nonstarch polysaccharides; S-AX: soluble arabinoxylans; S-NSP: soluble nonstarch polysaccharides; T-AX: total arabinoxylans; T-NSP: total nonstarch polysaccharides; X: xylanase enzyme.

^a–e^Means indicated with similar letters in columns do not differ significantly at *α* = 0.05.

**Table 4:** Insoluble nonstarch polysaccharides and arabinoxylan content in untreated, extruded, and hybrid-treated wheat flours.

**Sample**	**I-mannose (%)**	**I-galactose (%)**	**I-glucose (%)**	**I-arabinose (%)**	**I-xylose (%)**	**I-A/X (-)**
F	0.131 ± 0.002^a,b^	0.078 ± 0.003^a,b^	0.548 ± 0.024^a,b^	0.549 ± 0.019^a,b^	0.759 ± 0.019^a^	0.723 ± 0.007^a,b^
FC	0.133 ± 0.005^a,b^	0.074 ± 0.002^b^	0.535 ± 0.005^a,b^	0.458 ± 0.027^c,d^	0.674 ± 0.004^a,b^	0.680 ± 0.044^a,b^
FCX	0.142 ± 0.017^a,b^	0.081 ± 0.007^a,b^	0.528 ± 0.003^a,b^	0.518 ± 0.020^a,b,c^	0.651 ± 0.033^b^	0.798 ± 0.072^a^
EF23	0.129 ± 0.007^a,b^	0.078 ± 0.003^a,b^	0.541 ± 0.052^a,b^	0.474 ± 0.020^a,b,c^	0.647 ± 0.033^b^	0.733 ± 0.007^a,b^
EF25	0.144 ± 0.023^a,b^	0.080 ± 0.005^a,b^	0.522 ± 0.045^a,b^	0.468 ± 0.009^b,c,d^	0.634 ± 0.015^b^	0.739 ± 0.003^a,b^
EF27	0.120 ± 0.005^b^	0.074 ± 0.001^b^	0.573 ± 0.003^c^	0.507 ± 0.007^a,b,c^	0.694 ± 0.014^a,b^	0.731 ± 0.005^a,b^
EFC23	0.124 ± 0.005^b^	0.077 ± 0.002^a,b^	0.676 ± 0.004^b^	0.459 ± 0.011^c,d^	0.685 ± 0.035^a,b^	0.671 ± 0.050^b^
EFC25	0.162 ± 0.010^a^	0.098 ± 0.011^a^	0.565 ± 0.010*c*	0.550 ± 0.022^a^	0.760 ± 0.037^a^	0.724 ± 0.007^a,b^
EFC27	0.131 ± 0.014^a,b^	0.069 ± 0.014^b^	0.469 ± 0.052^d^	0.516 ± 0.078^a,b,c^	0.642 ± 0.043^b^	0.800 ± 0.068^a^
EFCX23	0.128 ± 0.001^b^	0.077 ± 0.004^a,b^	0.828 ± 0.018^a^	0.388 ± 0.000^d,e^	0.490 ± 0.015^c^	0.793 ± 0.023^a,b^
EFCX25	0.131 ± 0.014^a,b^	0.079 ± 0.002^a,b^	0.565 ± 0.038^c^	0.371 ± 0.003^e^	0.481 ± 0.035^c^	0.774 ± 0.050^a,b^
EFCX27	0.129 ± 0.003^b^	0.091 ± 0.016^a,b^	0.496 ± 0.028^a,b^	0.353 ± 0.013^e^	0.474 ± 0.057^c^	0.749 ± 0.063^a,b^

*Note*: *n* = 3.

Abbreviations: C: cellulase enzyme; E: extrusion treatment; F: flour; I-A/X: insoluble arabinose-to-xylose ratio; X: xylanase enzyme.

^a–e^Means indicated with similar letters in columns do not differ significantly at *α* = 0.05.

**Table 5:** Soluble nonstarch polysaccharides and arabinoxylan content in untreated, extruded, and hybrid treated wheat flours.

**Sample**	**S-mannose (%)**	**S-galactose (%)**	**S-glucose (%)**	**S-arabinose (%)**	**S-xylose (%)**	**S-A/X (-)**
F	0.265 ± 0.008^b,c,d^	0.118 ± 0.000^a,b^	0.352 ± 0.017^a,b^	0.276 ± 0.008^b^	0.328 ± 0.012^e^	0.842 ± 0.008^a^
FC	0.275 ± 0.001^a,b,c^	0.123 ± 0.003^a,b^	0.313 ± 0.012^a,b^	0.281 ± 0.002^b^	0.335 ± 0.003^e^	0.839 ± 0.000^a,b^
FCX	0.274 ± 0.006^a,b,c^	0.124 ± 0.002^a,b^	0.381 ± 0.005^a,b^	0.287 ± 0.001^b^	0.351 ± 0.007^e^	0.819 ± 0.013^a,b^
EF23	0.257 ± 0.010^c,d^	0.123 ± 0.003^a,b^	0.387 ± 0.066^a,b^	0.289 ± 0.000^b^	0.347 ± 0.005^e^	0.834 ± 0.012^a,b^
EF25	0.236 ± 0.025^d^	0.103 ± 0.008^b^	0.401 ± 0.069^a^	0.285 ± 0.007^b^	0.363 ± 0.038*d*^,e^	0.787 ± 0.062^a,b^
EF27	0.257 ± 0.001^c,d^	0.119 ± 0.000^a,b^	0.322 ± 0.000^a,b^	0.268 ± 0.008^b^	0.341 ± 0.007^e^	0.788 ± 0.005^a,b^
EFC23	0.257 ± 0.003^c,d^	0.152 ± 0.010^a^	0.328 ± 0.036^a,b^	0.256 ± 0.020^b^	0.338 ± 0.016^e^	0.758 ± 0.023^a,b^
EFC25	0.190 ± 0.010^e^	0.101 ± 0.015^b^	0.307 ± 0.003^b^	0.190 ± 0.009^c^	0.252 ± 0.010^f^	0.758 ± 0.066^a,b^
EFC27	0.300 ± 0.020^a^	0.121 ± 0.013^a,b^	0.369 ± 0.025^a,b^	0.283 ± 0.025^b^	0.401 ± 0.013^d^	0.708 ± 0.087^b^
EFCX23	0.291 ± 0.007^a,b^	0.139 ± 0.016^a^	0.360 ± 0.016^a,b^	0.402 ± 0.011^a^	0.541 ± 0.003^b^	0.742 ± 0.017^a,b^
EFCX25	0.245 ± 0.006^c,d^	0.105 ± 0.011^b^	0.382 ± 0.013^a,b^	0.387 ± 0.025^a^	0.489 ± 0.023^c^	0.793 ± 0.089^a,b^
EFCX27	0.262 ± 0.006^b,c,d^	0.104 ± 0.025^b^	0.328 ± 0.008^a,b^	0.418 ± 0.005^a^	0.590 ± 0.010^a^	0.709 ± 0.002^a,b^

*Note*: *n* = 3.

Abbreviations: C: cellulase enzyme; E: extrusion treatment; F: flour; S-A/X: soluble arabinose-to-xylose ratio; X: xylanase enzyme.

^a–u^Means indicated with similar letters in columns do not differ significantly at *α* = 0.05.

**Table 6:** Mixolab features of untreated, extruded, and hybrid-treated wheat flours.

**Sample**	**Hyd (%)**	**DT (min)**	**S (min)**	**C2 (nm)**	**C3 (nm)**	**C4 (nm)**	**C5 (nm)**
F	60.5 ± 0.1^g^	1.92 ± 0.19^d^	9.73 ± 0.12^a^	0.477 ± 0.006^d^	1.709 ± 0.010^a^	1.479 ± 0.006^a^	2.519 ± 0.001^a^
FC	60.6 ± 0.1^g^	2.66 ± 0.76^c,d^	9.60 ± 0.36^a^	0.432 ± 0.003^e,f^	1.673 ± 0.006^e^	1.433 ± 0.034^b^	2.414 ± 0.025^b^
FCX	61.2 ± 0.2^g^	3.22 ± 0.34^c^	9.37 ± 0.15^a^	0.423 ± 0.003^f^	1.674 ± 0.009^a^	1.351 ± 0.010^c^	2.173 ± 0.037^c^
EF23	117.7 ± 1.0^a^	1.91 ± 0.04^d^	2.60 ± 0.05^d^	0.452 ± 0.007^e^	0.474 ± 0.006^e^	0.326 ± 0.006^h^	0.543 ± 0.027^h^
EF25	106.6 ± 0.5^b^	2.38 ± 0.10^d^	3.76 ± 0.16^b,c^	0.526 ± 0.003^b^	0.664 ± 0.012^b,c,d^	0.497 ± 0.001^d^	0.801 ± 0.007^d,e^
EF27	94.3 ± 1.5^f^	2.59 ± 0.01^c,d^	3.83 ± 0.06^b^	0.524 ± 0.019^b^	0.745 ± 0.021^b^	0.491 ± 0.020^d,e^	0.844 ± 0.026^d^
EFC23	103.5 ± 0.4^c^	2.18 ± 0.29^d^	3.47 ± 0.06^b,c^	0.498 ± 0.009^c,d^	0.663 ± 0.009^b,c,d^	0.466 ± 0.005^d,e,f^	0.740 ± 0.034^f^
EFC25	99.3 ± 0.1^e^	2.49 ± 0.14^c,d^	3.71 ± 0.04^b,c^	0.521 ± 0.004^b^	0.656 ± 0.015^b,c,d^	0.459 ± 0.003^e,f^	0.753 ± 0.010^e,f^
EFC27	95.2 ± 0.1^f^	2.13 ± 0.20^d^	3.87 ± 0.06^b^	0.529 ± 0.003^b^	0.639 ± 0.099^c,d^	0.443 ± 0.002^f^	0.777 ± 0.006^e,f^
EFCX23	102.7 ± 0.3^c,d^	2.33 ± 0.15^d^	3.80 ± 0.10^b,c^	0.509 ± 0.003^b,c^	0.663 ± 0.006^b,c,d^	0.458 ± 0.002^e,f^	0.801 ± 0.006^d,e^
EFCX25	96.0 ± 0.5^f^	2.66 ± 0.04^c,d^	3.63 ± 0.12^b,c^	0.524 ± 0.010^b^	0.711 ± 0.009^b,c^	0.467 ± 0.005^d,e,f^	0.813 ± 0.012^d,e^
EFCX27	101.2 ± 0.3^d^	2.66 ± 0.01^c,d^	3.42 ± 0.03^c^	0.566 ± 0004^a^	0.576 ± 0.002^d^	0.366 ± 0.003^g^	0.657 ± 0.012^g^

*Note*: *n* = 3. C2: protein weakening; C3: starch gelatinization; C4: amylase activity; C5: starch retrogradation

Abbreviations: C: cellulase enzyme; DT: dough development time; E: extrusion treatment; F: flour; Hyd: hydration capacity; S: stability; X: xylanase enzyme.

^a–h^Means indicated with similar letters in columns do not differ significantly at *α* = 0.05.

**Table 7:** Pasting properties of untreated, extruded, and hybrid-treated wheat flours.

**Sample**	**Maximum viscosity (BU)**	**Trough viscosity (BU)**	**Final viscosity (BU)**	**Breakdown (BU)**	**Setback (BU)**	**Beginning of gelatinization (°C)**	**End of gelatinization (°C)**
F	745 ± 4^e^	208 ± 2^f^	583 ± 1^g^	537 ± 3^c^	376 ± 2^g^	60.2 ± 0.0^a^	86.6 ± 0.1^d^
FC	625 ± 2^h^	154 ± 1^i^	445 ± 1^i^	471 ± 2^d^	291 ± 1^i^	60.5 ± 0.0^a^	85.5 ± 0.0^f^
FCX	573 ± 1^i^	134 ± 1^j^	373 ± 4^j^	439 ± 2^e^	239 ± 3^j^	60.2 ± 0.1^a^	85.2 ± 0.1^g^
EF23	1057 ± 6^b^	366 ± 3^b^	765 ± 2^d^	692 ± 4^a^	399 ± 1^e^	31.1 ± 0.1^e^	86.2 ± 0.1^e^
EF25	1220 ± 9^a^	526 ± 5^a^	1073 ± 4^a^	694 ± 4^a^	547 ± 1^a^	42.4 ± 1.2^b^	88.0 ± 0.0^a^
EF27	649 ± 2^g^	229 ± 1^e^	649 ± 2^f^	421 ± 2^f^	420 ± 3^d^	38.2 ± 0.9^c,d^	86.7 ± 0.2^d^
EFC23	941 ± 16^c^	362 ± 5^b^	847 ± 10^b^	579 ± 11^b^	485 ± 5^b^	36.6 ± 2.0^c,d^	87.6 ± 0.1^b^
EFC25	689 ± 1^f^	246 ± 1^d^	670 ± 4^e^	443 ± 2^e^	424 ± 4^d^	35.4 ± 2.1^d^	86.9 ± 0.1^c^
EFC27	578 ± 8^i^	193 ± 2^g^	581 ± 5^g^	386 ± 6^g^	389 ± 3^f^	37.2 ± 0.8^c,d^	86.3 ± 0.1^e^
EFCX23	915 ± 4^d^	340 ± 11^c^	830 ± 8^c^	575 ± 7^b^	490 ± 3^b^	38.6 ± 1.1^c^	87.7 ± 0.2^b^
EFCX25	689 ± 5^f^	243 ± 9^d^	679 ± 3^e^	446 ± 4^e^	436 ± 6^c^	38.9 ± 0.8^c^	87.0 ± 0.1^c^
EFCX27	546 ± 8^j^	174 ± 2^h^	520 ± 4^h^	372 ± 6^g^	346 ± 2^h^	35.2 ± 0.6^d^	86.2 ± 0.1^e^

*Note*: *n* = 3.

Abbreviations: C: cellulase enzyme; E: extrusion treatment; F: flour; X: xylanase enzyme.

^a–j^Means indicated with similar letters in columns do not differ significantly at *α* = 0.05.

**Table 8:** Solvent retention capacity (SRC) values of untreated, extruded, and hybrid-treated wheat flours.

**Sample**	**SRCWa (%)**	**SRCSu (%)**	**SRCLa (%)**	**SRCSc (%)**	**GPI (-)**
F	70.005 ± 0.062^j^	114.973 ± 0.770^k^	118.294 ± 0.595^j^	87.839 ± 0.256^g^	0.583 ± 0.005^b^
FC	70.427 ± 0.049^j^	116.916 ± 1.228^j^	118.289 ± 0.624^j^	86.971 ± 0.080^g^	0.580 ± 0.001^c^
FCX	75.496 ± 0.278^i^	119.827 ± 0.876^i^	126.504 ± 0.950^i^	88.702 ± 0.153^g^	0.607 ± 0.003^a^
EF23	236.652 ± 0.530^a^	216.253 ± 0.725^a^	236.923 ± 0.276^a^	344.999 ± 2.037^a^	0.422 ± 0.002^f^
EF25	196.950 ± 0.530^d^	198.022 ± 0.477^b^	209.170 ± 0.385^b,c^	295.467 ± 2.609^b^	0.424 ± 0.002^e,f^
EF27	167.158 ± 0.227^h^	161.398 ± 0.224^h^	172.324 ± 0.952^h^	235.073 ± 1.585^f^	0.435 ± 0.001^c,d,e^
EFC23	193.667 ± 0.479^e^	189.778 ± 0.471^d^	202.480 ± 0.609^d^	276.106 ± 4.925^c^	0.435 ± 0.006^c,d,e^
EFC25	191.321 ± 0.869^f^	183.074 ± 0.305^e,f^	192.849 ± 2.034^f^	272.395 ± 6.960^c,d^	0.424 ± 0.011^e,f^
EFC27	177.957 ± 0.609^g^	166.261 ± 0.363^g^	183.706 ± 1.429^g^	248.603 ± 2.871^e^	0.443 ± 0.001^c^
EFCX23	203.601 ± 0.980^c^	191.850 ± 0.433^c^	206.880 ± 1.042^c^	291.260 ± 1.326^b^	0.428 ± 0.001^d,e,f^
EFCX25	195.729 ± 0.382^d^	182.101 ± 0.202^f^	198.613 ± 0.284^e^	264.583 ± 0.268^d^	0.445 ± 0.001^c^
EFCX27	216.286 ± 0.156^b^	184.618 ± 0.087^e^	210.365 ± 0.588^b^	295.415 ± 0.440^b^	0.438 ± 0.001^c,d^

*Note*: *n* = 3. La: 5% lactic acid; Sc: 5% sodium carbonate; Su: 50% sucrose; Wa: distilled water.

Abbreviations: C: cellulase enzyme; E: extrusion treatment; F: flour; GPI: Gluten Performance Index; SRC: solvent retention capacity; X: xylanase enzyme.

^a–k^Means indicated with similar letters in columns do not differ significantly at *α* = 0.05.

## Data Availability

All data are included in this paper.

## References

[1] Alam S. A., Järvinen J., Kirjoranta S., Jouppila K., Poutanen K., Sozer N. (2014). Influence of Particle Size Reduction on Structural and Mechanical Properties of Extruded Rye Bran. *Food and Bioprocess Technology*.

[2] Deng F., Hu X., Wang Y., Luo S., Liu C. (2023). Improving the Yield of Feruloyl Oligosaccharides From Rice Bran Through Enzymatic Extrusion and Its Mechanism. *Food*.

[3] Guy R. (2001). *Extrusion Cooking: Technology and Application*.

[4] Valentina S., Paul A., Andrew P., Senol L. (2010). The Advantage of Using Extrusion Processing for Increasing Dietary Fibre Level in Gluten-Free Products. *Food Chemistry*.

[5] Leonard W., Zhang P., Ying D., Fang Z. (2020). Application of Extrusion Technology in Plant Food Processing Byproducts: An Overview. *Comprehensive Reviews in Food Science and Food Safety*.

[6] Wójtowicz A., Oniszczuk A., Oniszczuk T. (2017). Application of Moldavian Dragonhead (Dracocephalum moldavica L.) Leaves Addition as a Functional Component of Nutritionally Valuable Corn Snacks. *Journal of Food Science and Technology*.

[7] Contreras-Jiménez B., Gaytán-Martínez M., Morales-Sánchez E. (2017). Effects of Tempering Time, Ca(OH)_2_ Concentration, and Particle Size on the Rheological Properties of Extruded Corn Flour. *Cereal Chemistry*.

[8] Moscicki L. (2011). Extrusion-Cooking Techniques. *Application, Theory and Sustainability*.

[9] Bouasla A., Wójtowicz A., Zidoune M. N. (2016). Gluten-Free Precooked Rice-Yellow Pea Pasta: Effect of Extrusion-Cooking Conditions on Phenolic Acids Composition, Selected Properties and Microstructure. *Journal of Food Science*.

[10] Combrzyński M., Oniszczuk T., Wójtowicz A. (2023). Nutritional Characteristics of New Generation Extruded Snack Pellets With Edible Cricket Flour Processed at Various Extrusion Conditions. *Antioxidants*.

[11] Kręcisz M., Wójtowicz A. (2017). Evaluation of Selected Properties of Gluten-Free Instant Gruels Processed Under Various Extrusion-Cooking Conditions. *Acta Scientiarum Polonorum Technologia Alimentaria*.

[12] Mitrus M., Wójtowicz A., Kocira S. (2020). Effect of Extrusion-Cooking Conditions on the Pasting Properties of Extruded White and Red Bean Seeds. *International Agrophysics*.

[13] Wójtowicz A., Combrzyński M., Biernacka B. (2023). Application of Edible Insect Flour as a Novel Ingredient in Fortified Snack Pellets: Processing Aspects and Physical Characteristics. *Pro*.

[14] Wang H., van der Berg F. W. J., Zhang W. (2022). Differences in Physicochemical Properties of High-Moisture Extrudates Prepared From Soy and Pea Protein Isolates. *Food Hydrocolloids*.

[15] Zahari I., Ferawati F., Helstad A. (2020). Development of High-Moisture Meat Analogues With Hemp and Soy Protein Using Extrusion Cooking. *Food*.

[16] Fischer T. (2004). Effect of Extrusion Cooking on Protein Modification in Wheat Flour. *European Food Research and Technology*.

[17] Robin F., Dubois C., Pineau N., Labat E., Théoduloz C., Curti D. (2012). Process, Structure and Texture of Extruded Whole Wheat. *Journal of Cereal Science*.

[18] Zhou X., Xing Y., Meng T. (2021). Preparation of V-Type Cold Water-Swelling Starch by Ethanolic Extrusion. *Carbohydrate Polymers*.

[19] Aktas-Akyildiz E., Masatcioglu M. T., Köksel H. (2020). Effect of Extrusion Treatment on Enzymatic Hydrolysis of Wheat Bran. *Journal of Cereal Science*.

[20] Chen Y., Ye R., Yin L., Zhang N. (2014). Novel Blasting Extrusion Processing Improved the Physicochemical Properties of Soluble Dietary Fiber From Soybean Residue and In Vivo Evaluation. *Journal of Food Engineering*.

[21] Singkhornart S., Lee S. G., Ryu G. H. (2013). Influence of Twin-Screw Extrusion on Soluble Arabinoxylans and Corn Fiber Gum From Corn Fiber. *Journal of the Science of Food and Agriculture*.

[22] Dang T. T., Vasanthan T. (2019). Modification of Rice Bran Dietary Fiber Concentrates Using Enzyme and Extrusion Cooking. *Food Hydrocolloids*.

[23] Yan X., Ye R., Chen Y. (2015). Blasting Extrusion Processing: The Increase of Soluble Dietary Fiber Content and Extraction of Soluble-Fiber Polysaccharides From Wheat Bran. *Food Chemistry*.

[24] Cervantes-Ramírez J., Cabrera-Ramirez A. H., Morales-Sánchez E. (2020). Amylose-Lipid Complex Formation From Extruded Maize Starch Mixed With Fatty Acids. *Carbohydrate Polymers*.

[25] Yağcı S., Kocabaş D. S., Çalışkanb R., Özbek H. N. (2022). Statistical Investigation of the Bioprocess Conditions of Alkali Combined Twin-Screw Extrusion Pretreatment to Enhance Fractionation and Enzymatic Hydrolysis of Bulgur Bran. *Journal of the Science of Food and Agriculture*.

[26] Gao Y., Xia H., Pramudya A., Melo E., Dugmore T., Matharu A. (2019). Defibrillated Celluloses via Dual Twin-Screw Extrusion and Microwave Hydrothermal Treatment of Spent Pea Biomass. *ACS Sustainable Chemistry & Engineering*.

[27] Zhang Y., Li T., Shen Y. (2020). Extrusion Followed by Ultrasound as a Chemical-Free Pretreatment Method to Enhance Enzymatic Hydrolysis of Rice Hull for Fermentable Sugars Production. *Industrial Crops and Products*.

[28] Zeng Z., Li Y., Yang R. (2017). The Relationship Between Reducing Sugars and Phenolic Retention of Brown Rice After Enzymatic Extrusion. *Journal of Cereal Science*.

[29] Román L., Martínez M., Rosell C., Gómez M. (2017). Changes in Physicochemical Properties and In Vitro Starch Digestion of Native and Extruded Maize Flours Subjected to Branching Enzyme and Maltogenic *α*-Amylase Treatment. *International Journal of Biological Macromolecules*.

[30] Kong C., Duan C., Zhang S., Liu R., Sun Y., Zhou S. (2023). Effects of Co-Modification by Extrusion and Enzymatic Hydrolysis on Physicochemical Properties of Black Wheat Bran and Its Prebiotic Potential. *Food*.

[31] De Pilli T., Legrand J., Giuliani R., Derossi A., Severini C. (2009). Effect of Processing Variables and Enzymatic Activity on Wheat Flour Dough Extruded Under Different Operating Conditions. *Food Technology and Biotechnology*.

[32] Qi X., Li Y., Zhang W. (2024). Proteolysis Improves the Foaming Properties of Rice Protein Fibrils: Structure, Physicochemical Properties Changes, and Application in Angel Food Cake. *Food Chemistry*.

[33] Uthumporn U., Shariffa Y. N., Karim A. A. (2012). Hydrolysis of Native and Heat-Treated Starches at Sub-Gelatinization Temperature Using Granular Starch Hydrolyzing Enzyme. *Applied Biochemistry and Biotechnology*.

[34] de Souza T., Kawaguti H. Y. (2021). Cellulases, Hemicellulases, and Pectinases: Applications in the Food and Beverage Industry. *Food and Bioprocess Technology*.

[35] Chen H., Chen Z., Fu Y. (2019). Structure, Antioxidant, and Hypoglycemic Activities of Arabinoxylans Extracted by Multiple Methods From Triticale. *Antioxidants*.

[36] Zhou S., Liu X., Guo Y., Wang Q., Peng D., Cao L. (2010). Comparison of the Immunological Activities of Arabinoxylans From Wheat Bran With Alkali and Xylanase-Aided Extraction. *Carbohydrate Polymers*.

[37] Izydorczyk M., Eliasson A. C. (2017). Functional Properties of Cereal Cell Wall Polysaccharides. *Carbohydrates in Food*.

[38] Barron C., Bar-L'Helgouac'h C., Champ M., Saulnier L. (2020). Arabinoxylan Content and Grain Tissue Distribution Are Good Predictors of the Dietary Fibre Content and Their Nutritional Properties in Wheat Products. *Food Chemistry*.

[39] Zannini E., Bravo Núñez A., Sahin A., Arendt E. (2022). Arabinoxylans as Functional Food Ingredients: A Review. *Foods*.

[40] Kaur A., Yadav M., Singh B., Bhinder S., Simon S., Singh N. (2019). Isolation and Characterization of Arabinoxylans From Wheat Bran and Study of Their Contribution to Wheat Flour Dough Rheology. *Carbohydrate Polymers*.

[41] Lewko P., Wójtowicz A., Gancarz M. (2023). Distribution of Arabinoxylans and Their Relationship With Physiochemical and Rheological Properties in Wheat Flour Mill Streams as an Effective Way to Predict Flour Functionality. *Applied Sciences*.

[42] (2009). *Approved Method of the AACC*.

[43] McCleary B., DeVries J., Rader J. (2012). Determination of Insoluble, Soluble, and Total Dietary Fiber (CODEX Definition) by Enzymatic-Gravimetric Method and Liquid Chromatography: Collaborative Study. *Journal of AOAC International*.

[44] Englyst H., Cummings J. (1984). Simplified Method for the Measurement of Total Non-Starch Polysaccharides by Gas-Liquid Chromatography of Constituent Sugars as Alditol Acetates. *Analyst*.

[45] Herlich K. (1990). *AOAC Official Methods of Analysis*.

[46] Fraś A. (2011). *Analysis of the Variability of the Content of Dietary Fiber and Alkylresorcinols in Common Wheat Grain (Triticum aestivum L.)*.

[47] Dubat A., Rosell C. M., Gallagher E. (2013). *Mixolab: A New Approach to Rheology*.

[48] (2018). *ICC Standard Methods*.

[49] Rani M., Singh G., Siddiqi R. A., Gill B. S., Sogi D. S., Bhat M. A. (2021). Comparative Quality Evaluation of Physicochemical, Technological, and Protein Profiling of Wheat, Rye, and Barley Cereals. *Frontiers in Nutrition*.

[50] Kweon M., Slade L., Levine H. (2011). Solvent Retention Capacity (SRC) Testing of Wheat Flour: Principles and Value in Predicting Flour Functionality in Different Wheat-Based Food Processes and in Wheat Breeding—A Review. *Cereal Chemistry*.

[51] Tomaszewska E., Rudyk H., Dobrowolski P. (2021). Changes in the Intestinal Histomorphometry, the Expression of Intestinal Tight Junction Proteins, and the Bone Structure and Liver of Pre-Laying Hens Following Oral Administration of Fumonisins for 21 Days. *Toxins*.

[52] Combrzyński M., Oniszczuk T., Kupryaniuk K. (2021). Physical Properties, Spectroscopic, Microscopic, X-Ray, and Chemometric Analysis of Starch Films Enriched With Selected Functional Additives. *Materials*.

[53] Rabiej M. (2017). Application of the Particle Swarm Optimization Method for the Analysis of Wide-Angle X-Ray Diffraction Curves of Semicrystalline Polymers. *Journal of Applied Crystallography*.

[54] Yoo S.-H., Jane J.-L. (2002). Structural and Physical Characteristics of Waxy and Other Wheat Starches. *Carbohydrate Polymers*.

[55] Moreno-Rivas S. C., Medina-Rodríguez C. L., Torres-Chávez P. I., Ramírez-Wong B., Platt-Lucero L. C. (2014). Changes in the Solubility of Corn Proteins Through Interaction With the Arabinoxylans in Extruded Nixtamalized Corn Flour Treated With Xylanase. *Plant Foods for Human Nutrition*.

[56] Ma F., Li X., Yin J., Ma L., Li D. (2020). Optimisation of Double-Enzymatic Extraction of Arabinoxylan From Fresh Corn Fibre. *Journal of Food Science and Technology*.

[57] Ye G., Wu Y., Wang L. (2021). Comparison of Six Modification Methods on the Chemical Composition, Functional Properties and Antioxidant Capacity of Wheat Bran. *LWT*.

[58] Arcila J., Weier S., Rose D. (2015). Changes in Dietary Fiber Fractions and Gut Microbial Fermentation Properties of Wheat Bran After Extrusion and Bread Making. *Food Research International*.

[59] Fadel A., Mahmoud A., Ashworth J., Li W., Ng Y. L., Plunkett A. (2018). Health-Related Effects and Improving Extractability of Cereal Arabinoxylans. *International Journal of Biological Macromolecules*.

[60] Long D., Ye F., Zhao G. (2014). Optimization and Characterization of Wheat Bran Modified by In Situ Enhanced CO_2_ Blasting Extrusion. *LWT-Food Science and Technology*.

[61] Gartaula G., Dhital S., Netzel G. (2018). Quantitative Structural Organisation Model for Wheat Endosperm Cell Walls: Cellulose as an Important Constituent. *Carbohydrate Polymers*.

[62] Santala O., Nordlund E., Poutanen K. (2013). Use of an Extruder for Pre-Mixing Enhances Xylanase Action on Wheat Bran at Low Water Content. *Bioresource Technology*.

[63] Andersson A., Andersson R., Jonsall A., Andersson J., Fredriksson H. (2017). Effect of Different Extrusion Parameters on Dietary Fiber in Wheat Bran and Rye Bran. *Journal of Food Science*.

[64] Hell J., Donaldson L., Michlmayr H. (2015). Effect of Pretreatment on Arabinoxylan Distribution in Wheat Bran. *Carbohydrate Polymers*.

[65] Demuth T., Betschart J., Nyström L. (2020). Structural Modifications to Water-Soluble Wheat Bran Arabinoxylan Through Milling and Extrusion. *Carbohydrate Polymers*.

[66] Martínez M., Oliete B., Gómez M. (2013). Effect of the Addition of Extruded Wheat Flours on Dough Rheology and Bread Quality. *Journal of Cereal Science*.

[67] Pasqualone A., Costantini M., Labarbuta R., Summo C. (2021). Production of Extruded-Cooked Lentil Flours at Industrial Level: Effect of Processing Conditions on Starch Gelatinization, Dough Rheological Properties and Techno-Functional Parameters. *LWT*.

[68] Devi A., Sindhu R., Khatkar B. S. (2019). Morphological, Pasting, and Textural Characterization of Starches and Their Sub Fractions of Good and Poor Cookie Making Wheat Varieties. *Journal of Food Science and Technology*.

[69] Liu S., Du C., Feng J. (2023). Characterization of Starch Physicochemical Properties and Grain Transcriptome Reveal the Mechanism for Resistant Starch Accumulation. *Agronomy*.

[70] Zeng J., Li G., Gao H., Ru Z. (2011). Comparison of A and B Starch Granules From Three Wheat Varieties. *Molecules*.

[71] Lewko P., Wójtowicz A., Kamiński D. M. (2024). The Influence of Processing Using Conventional and Hybrid Methods on the Composition, Polysaccharide Profiles and Selected Properties of Wheat Flour Enriched With Baking Enzymes. *Food*.

[72] Saiah R., Sreekumar P. A., Leblanc N., Castandet M., Saiter J.-M. (2007). Study of Wheat-Flour-Based Agropolymers: Influence of Plasticizers on Structure and Aging Behavior. *Cereal Chemistry*.

[73] Leblanc N., Saiah R., Beucher E., Gattin R., Castandet M., Saiter J.-M. (2008). Structural Investigation and Thermal Stability of New Extruded Wheat Flour Based Polymeric Materials. *Carbohydrate Polymers*.

[74] Oliveira L. C., Barros J. H. T., Rosell C. M., Steel C. J. (2017). Physical and Thermal Properties and X-Ray Diffraction of Corn Flour Systems as Affected by Whole Grain Wheat Flour and Extrusion Conditions. *Starch‐Stärke*.

[75] Jafari M., Koocheki A., Milani E. (2017). Effect of Extrusion Cooking on Chemical Structure, Morphology, Crystallinity and Thermal Properties of Sorghum Flour Extrudates. *Journal of Cereal Science*.

[76] Liu Y., Chen J., Luo S. (2017). Physicochemical and Structural Properties of Pregelatinized Starch Prepared by Improved Extrusion Cooking Technology. *Carbohydrate Polymers*.

[77] Enríquez-Castro C. M., Torres-Chávez P. I., Ramírez-Wong B. (2020). Physicochemical, Rheological, and Morphological Characteristics of Products From Traditional and Extrusion Nixtamalization Processes and Their Relation to Starch. *International Journal of Food Science*.

[78] Alam M. S., Kaur J., Khaira H., Gupta K. (2016). Extrusion and Extruded Products: Changes in Quality Attributes as Affected by Extrusion Process Parameters: A Review. *Critical Reviews in Food Science and Nutrition*.

